# Efficacy and safety of robot-assisted core decompression for osteonecrosis of the femoral head: a meta-analysis of single-arm studies

**DOI:** 10.1186/s12891-026-09777-y

**Published:** 2026-05-06

**Authors:** Huan Liu, Dawei Jiang, Feng Wang, Zheming Bao, Hao Xing, Bo Wu

**Affiliations:** Department of Orthopaedics, The 960th Hospital of PLA, 25 shifan Road, Tianqiao District, Jinan, Shandong 250031 China

**Keywords:** Femoral head necrosis, Robot-assisted surgery, Core decompression, Meta-analysis, Systematic review

## Abstract

**Objective:**

Osteonecrosis of the femoral head (ONFH) is a common disabling hip disorder with an annually increasing incidence due to factors such as corticosteroid administration. Core decompression is an effective surgical intervention for early-stage ONFH, and robot-assisted technology can enhance the accuracy and minimally invasiveness of this procedure. However, its clinical efficacy requires comprehensive evaluation with high-quality evidence. This study aimed to assess the clinical efficacy and safety of robot-assisted core decompression (RACD) in the treatment of ONFH.

**Methods:**

We systematically searched Chinese and English databases up to December 1, 2025, for case series, cohort studies, and randomized controlled trials (RCTs) on RACD for ONFH. The primary outcome was the femoral head collapse rate; secondary outcomes included the Harris Hip Score (HHS), Visual Analogue Scale (VAS) score, and postoperative complications. Meta-analysis was performed using fixed-effects or random-effects models, with subgroup and sensitivity analyses conducted to verify results.

**Results:**

A total of 12 studies were included (1 RCT, 9 cohort studies, 2 case series), involving 325 patients. Meta-analysis showed that the overall femoral head collapse rate after robot-assisted core decompression was 9% (95% CI: 7%–13%). In the subgroup of ARCO Stage II patients with follow-up > 1 year, the femoral head collapse rate was 12% (95% CI: 8%–17%). The mean postoperative improvement in HHS was 17.38 points (95% CI: 14.40–20.37), and the mean postoperative improvement in VAS score was 2.75 points (95% CI: 2.01–3.50). No complications such as infection or neurovascular injury were reported in any of the studies that reported complications (*n* = 7, 222 patients in total). Sensitivity analysis indicated that the results were robust. Egger’s test suggested potential publication bias for HHS, VAS, and femoral head collapse rate; however, the adjusted effect sizes still supported clinical benefit. Given the high heterogeneity, the adjusted effect sizes should be interpreted with caution.

**Conclusion:**

Available evidence supports the effectiveness of robot-assisted core decompression in the treatment of early-stage ONFH. RACD yields functional improvement and pain relief in patients with ONFH, with acceptable short-to-medium-term safety. Current evidence, however, are insufficient to confirm that RACD yields superior hip preservation efficacy compared with MCD. Further direct, high-quality comparative studies are required to determine and quantify the benefits of RACD compared to MCD.

**Trial registration:**

PROSPERO (CRD420251266114).

**Supplementary Information:**

The online version contains supplementary material available at 10.1186/s12891-026-09777-y.

## Introduction

Osteonecrosis of the femoral head (ONFH) is characterized by ischemic necrosis of bone tissue secondary to impaired local blood supply. It is a common refractory and disabling condition in orthopedics, and one of the leading causes of hip pain and dysfunction in adults [1]. With the widespread use of glucocorticoids, the incidence of ONFH has risen considerably, attracting increasing clinical attention [2]. In the United States, the annual number of new ONFH cases is estimated to range from 10,000 to 30,000 [3]. As of 2015, the cumulative number of patients with non-traumatic ONFH in China was approximately 8.12 million, and the actual prevalence may be even higher [4]. Recent studies have further indicated that ONFH accounts for 36.2% of all osteonecrosis cases [5], underscoring the substantial public health burden of this disease.

Common risk factors, including glucocorticoid use, alcohol abuse, and hip trauma, can disrupt the intraosseous blood supply of the femoral head, leading to the necrosis of bone tissue and marrow components, followed by a series of repair processes [6]. These pathological changes may cause bone structural damage and subchondral fractures, which gradually progress to femoral head collapse and deformation, subsequent joint destruction, and eventually secondary osteoarthritis [7]. Moreover, ONFH predominantly affects young and middle-aged adults, severely impairing their daily living and work capacity [8]. Without timely and effective intervention, more than 90% of patients may develop femoral head collapse within 5 years, eventually requiring total hip arthroplasty (THA) [9, 10]. Therefore, early diagnosis and treatment are crucial for the management of early-stage ONFH.

Core decompression is a core clinical treatment strategy for ONFH, and is widely accepted in clinical practice due to its minimally invasive nature and ability to rapidly alleviate postoperative pain [11]. In recent years, the application of robot-assisted technology has further improved the minimally invasiveness and precision of core decompression, and relevant clinical efficacy studies have been increasingly reported.

In addition to single-arm efficacy evaluations, several studies have attempted to directly compare robot-assisted core decompression (RACD) with conventional manual core decompression (MCD).

For example, retrospective cohort studies by Luo et al. [12] and Bi et al. [13] demonstrated that RACD may be superior to MCD in terms of surgical accuracy, reduced intraoperative fluoroscopy frequency, and minimized surgical trauma. A meta-analysis by Ouyang et al. [10] also synthesized partial comparative data and suggested a similar trend. However, these comparative studies are generally limited by small sample sizes, short follow-up durations, and non-randomized study designs, resulting in low evidence strength. Furthermore, the femoral head collapse rate—an important endpoint for treatment failure—still requires more in-depth investigation.

More importantly, there is currently a lack of systematic quantitative summaries focusing specifically on the efficacy and safety of RACD itself. This is essential for accurately evaluating the clinical value of this technique and providing key parameters (e.g., effect size, event rate) for the design of high-quality RCTs in the future. Therefore, at the current stage with scarce high-quality head-to-head comparative evidence, conducting a systematic review and meta-analysis of existing single-arm studies on RACD has important transitional and bridging significance.

This study aimed to perform a systematic review and meta-analysis by synthesizing data from existing single-arm studies to quantitatively evaluate the efficacy and safety of RACD in the treatment of ONFH. The findings are expected to provide orthopedic clinicians with a clearer and more intuitive understanding of the therapeutic advantages of RACD, as well as reference parameters for designing future high-quality clinical studies.

## Methods

### Registration and reporting

This study was registered in the PROSPERO database (Registration ID: CRD420251266114). The conduct and reporting of this systematic review strictly adhered to the Preferred Reporting Items for Systematic Reviews and Meta-Analyses (PRISMA) 2020 guidelines [14], and the PRISMA 2020 Checklist is provided as supplementary material.

In addition, we would like to provide some clarification. As femoral head collapse rate was the primary outcome measure in this study, we performed an additional post-hoc analysis to provide clinical reference. Specifically, we extracted corresponding data from MCD for this exploratory analysis.

### Literature search

Detailed information on the search strategy is provided in Supplementary Material 1. We systematically searched multiple databases for studies on RACD for ONFH, including Chinese databases (China National Knowledge Infrastructure [CNKI], WanFang Data, VIP Chinese Journal Database, Chinese Biomedical Literature Database [CBM]) and English databases (PubMed, Embase, Cochrane Library, Web of Science). Unpublished grey literature was also included. The search was conducted up to December 1, 2025.

The search strategy combined subject headings and free-text terms, with core keywords including “robot-assisted”, “femoral head necrosis”, and “core decompression”. Additionally, we manually screened the reference lists of included studies and relevant meta-analyses to identify potentially overlooked eligible studies.

### Inclusion and exclusion criteria

This study included studies that systematically reported the clinical efficacy of RACD for ONFH. The primary study types were case series, cohort studies, and the RACD arm of RCTs.

Population (P): Patients with a confirmed diagnosis of ONFH.

Intervention (I): Treatment with RACD.

Control (C): No traditional control group was set for the main analysis, which aimed to synthesize single-arm efficacy data of RACD. For clinical contextualization, data from the MCD arms concurrently reported in included cohort studies and RCTs were extracted for indirect comparison or historical reference analysis.

Outcomes (O):

Primary outcome: The femoral head collapse rate after RACD was defined as the proportion of patients with disease progression to ARCO Stage III or above on imaging (X-ray or CT) at the final follow-up (calculated as the number of samples with femoral head collapse divided by the total number of samples undergoing RACD).

Secondary outcomes: Postoperative improvements in HHS and VAS scores, and the incidence of surgery-related complications.

No language restrictions were applied during study screening.

Exclusion criteria:

(1) Studies without extractable relevant outcome data; (2) Duplicate publications; (3) Studies using only computer navigation without physical robotic arm assistance; (4) Studies with missing follow-up data exceeding 20%; (5) Studies including patients with pre-existing femoral head collapse.

### Data extraction

Literature screening and data extraction were independently performed by two investigators following a three-stage process (title → abstract → full text). Bibliographic records were managed using EndNote v21 reference management software, and extracted data were uniformly archived in a structured electronic spreadsheet for preliminary analysis. Disagreements on study inclusion were resolved through joint re-evaluation; if consensus could not be reached, a third methodological expert was consulted for arbitration. The screening process strictly followed the pre-defined inclusion and exclusion criteria, and the extracted study characteristics (first author, publication year, region, study design, participant cohort features) were all subjected to consistency verification.

### Quality assessment

Case series were critically appraised using the JBI Critical Appraisal Checklist for Case Series [15] (10 appraisal items). Cohort studies were assessed using the Newcastle–Ottawa Scale (NOS) [16], with a total score of 9 points (4 for participant selection, 2 for group comparability, 3 for exposure/outcome assessment). RCTs were evaluated using the Cochrane Collaboration’s revised Risk of Bias 2 (RoB 2) tool [17], with judgments of “low risk”, “some concerns”, or “high risk” for five core domains (randomization process, deviations from intended interventions, missing outcome data, outcome measurement, selective reporting), based on which an overall risk of bias was determined. Inter-rater reliability was assessed using the weighted Cohen’s kappa coefficient. Any disagreements during the quality assessment process were resolved by consulting a third reviewer.

### Statistical analysis

Meta-analysis was performed using R software (Version 4.4.3) with the Meta package, and effect sizes (ES) and 95% CIs were synthesized using rigorous methodologies. Binary variables were presented as counts and percentages, and continuous variables as means and standard deviations (SDs). A random-effects model based on the generalized linear mixed model (GLMM) was used to pool proportions, which was fitted on the logit scale to effectively stabilize variance and handle extreme proportions.

Since the included studies did not report change scores for continuous variables (HHS, VAS) and no patient loss to follow-up was observed, we calculated the SD of changes from baseline using the formula in Sect. 6.5.2.8 (Input of SDs for changes from baseline) of the *Cochrane Handbook for Systematic Reviews of Interventions* (https://china.cochrane.org/resources/cochrane-resources/cochrane-handbook), with a preset correlation coefficient of 0.5 for the primary analysis.

$$\mathrm{Corr}_{E}=\frac{SD_{E,baseline}^{2}+SD_{E,final}^{2}-SD_{E,change}^{2}}{2\times{SD_{E,baseline\times{SD_{E,final}}}}}$$

Specifically, the metamean() function was used to pool the calculated change scores, and restricted maximum likelihood estimation (REML) was employed to estimate between-study heterogeneity. Sensitivity analyses were further conducted by substituting correlation coefficients of 0.25 and 0.75 into the formula to calculate the SD of changes, aiming to verify the robustness of the results.

Publication bias was assessed using Egger’s regression asymmetry test with a two-tailed α = 0.05 (*P* < 0.05 indicated statistically significant publication bias). If publication bias was detected, the trim-and-fill method (trimfill() function) was applied to re-estimate the pooled effect size. Model selection was based on the I² statistic for heterogeneity: a fixed-effects model was used when I² ≤ 50%, and a random-effects model when I² > 50%. The robustness of the results was systematically verified via leave-one-out sensitivity analysis (metainf() function), which iteratively excluded individual studies and recalculated the overall estimate. For outcome measures unsuitable for quantitative analysis, a narrative synthesis was conducted for systematic assessment.

As femoral head collapse rate was the primary outcome measure in this study, to provide clinical reference, we extracted corresponding data from MCD for exploratory analysis. Specifically, we additionally calculated the femoral head collapse rate following MCD. Furthermore, we performed a head-to-head meta-analysis comparison of femoral head collapse.

For the femoral head collapse rate after MCD, we pooled proportions using a random-effects model based on the GLMM, fitted on the logit scale. For the head-to-head meta-analysis comparison of femoral head collapse, considering the common presence of sparse data, we still selected the GLMM to derive the pooled OR and 95% CI.

All data were directly extracted from the original literature, and no additional data transformation or imputation was performed.

### Subgroup analysis

Subgroup analyses were performed for all outcome measures according to ONFH ARCO stage, follow-up duration, and bone graft material type, where data were available.

### Meta-regression

Meta-regression analyses (metareg() function) were conducted to explore potential sources of heterogeneity, with covariates including mean follow-up duration (months), mean age, ONFH ARCO stage, and bone graft material type. Univariate meta-regression was performed first, and covariates with statistically significant associations were then included in a multivariate meta-regression model.

### Certainty of evidence

We will assess the levels of evidence in accordance with The Oxford 2011 Levels of Evidence (http://www.cebm.net/index.aspx?o=5653). Specifically, we evaluate the incidence of femoral head collapse and complications using the evidence assessment criteria for the question How common is the problem?. We assess the improvements in HSS and VAS scores using the evidence assessment criteria for the question Does this intervention help? (Treatment Benefits).

## Results

### Literature search and screening process

**Fig. 1:**
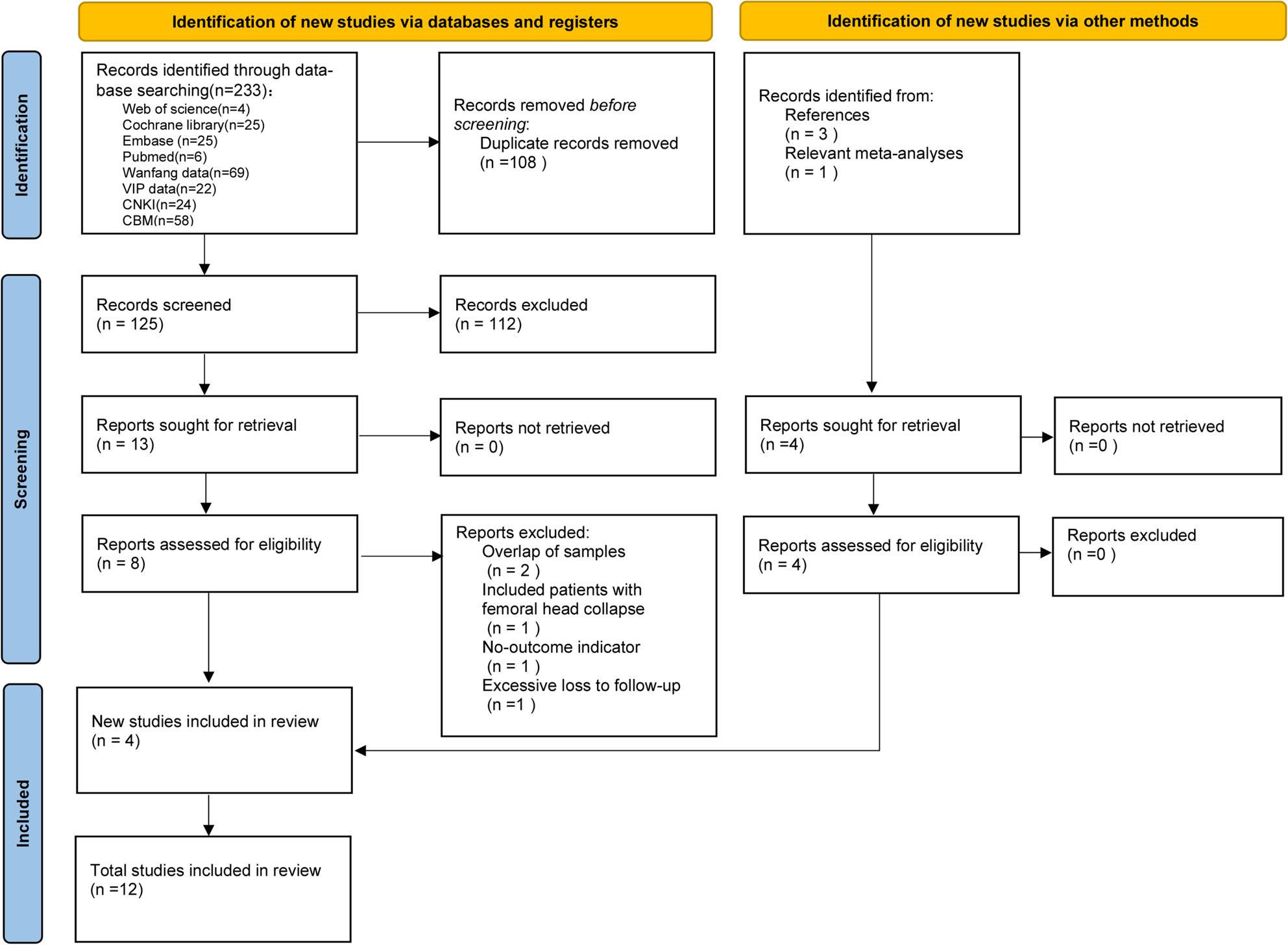
PRISMA 2020 flow diagram of study selection process. A total of 233 records were identified through database searching. After removing 108 duplicates, 120 records remained. Following title and abstract screening, 112 records were excluded, leaving 13 reports sought for retrieval. All 13 reports were successfully retrieved and assessed for eligibility. Five reports were excluded due to sample overlap (*n*=2), inclusion of patients with femoral head collapse (*n*=1), absence of outcome indicators (*n*=1), and excessive loss to follow-up (*n*=1). An additional 4 eligible studies were identified through citation searching of included articles and relevant reviews. Ultimately, 12 studies were included in the meta-analysis

A total of 228 records were initially identified from the database search. After removing 108 duplicates, 120 unique records were screened based on titles and abstracts, and 13 studies were preliminarily selected. Full-text review led to the exclusion of 5 studies for the following reasons: 2 with overlapping samples with subsequent related research [18, 19], 1 lacking relevant outcome measures [20], 1 including patients with pre-existing femoral head collapse [21], and 1 with excessive missing follow-up data [22]. To minimize publication bias, the reference lists of included studies and relevant meta-analyses were manually screened, identifying 4 additional eligible studies. Ultimately, 12 studies were included in the final analysis [12, 13, 23–32]. The complete literature screening process is illustrated in Fig. 1.

In addition, for potentially overlapping studies, we will assess them based on three criteria: consistency in research topic, consistency in research institution, and consistency in research team. When two studies meet all three criteria above, they will be regarded as duplicate studies, and the one with a smaller sample size will be excluded. Detailed information on excluded studies is provided in Supplementary Material 2.

### Characteristics of included studies

A total of 12 studies [12, 13, 23–32] involving 325 patients who underwent RACD were included, consisting of 1 RCT [31], 9 cohort studies (including 1 grey literature publication [25]) [12, 13, 25–30, 32], and 2 case series [23, 24]. The detailed characteristics of the included studies are summarized in Table 1.

**Table 1:** Characteristics of the included studies

Study	Study Design	Stage	Country	M/F	Mean age(year)	Sample size (No.patients /No.hips)	Average follow-up time	Bone Grafting Materials	Bone Grafting Materials	Bone Graft Classification Groups	Orthopedic Robot
Zhao2018 [23]	case series	ARCOⅡ	China	7/2	44.6	9/16	12.7 months	Autogenous cancellous bone and allogeneic cancellous bone	Drilling into the necrotic lesion area and removing the cylindrical bone core	Bio+	GD-2000 Orthopedic Surgical Robot Navigation and Positioning System (TINAVI Medical Technologies Co., Ltd., Beijing)
Bi2019 [13]	cohort study	ARCOⅡ	China	7/2	34.88	9/16	26.4 months	Bone grafting was performed without specifying the material used.	Drilling into the necrotic lesion area and removing the cylindrical bone core	Unclear	TiRobot™ (TI NAVI Medical Technologies Co., Ltd., Beijing, China)
Luo2020 [12, 18﻿]	cohort study	Ficat I and Ficat II	China	18/12	53	30/41	6months	It was not specified whether bone grafting was performed.	Drilling into the necrotic lesion area and removing the cylindrical bone core	Unclear	TiRobot™ (TI NAVI Medical Technologies Co., Ltd., Beijing, China)
Zhang2022 [19, 26]	cohort study	ARCOⅠ	China	19/11	38.5	30/30	23.35months	It was not specified whether bone grafting was performed.	Multiple percutaneous drillings	Unclear	TiRobot™ (TI NAVI Medical Technologies Co., Ltd., Beijing, China)
Yang2022 [24]	case series	ARCOⅡ	China	14/4	42.6	18/26	18.6months	Human umbilical cord Mesenchymal Stem Cells (hUC-MSCs), autogenous cancellous bone granules, and calcium sulfate (CaSO₄)-calcium phosphate (CaPO₄) bone graft substitutes.	Drilling into the necrotic lesion area and removing the cylindrical bone core	Bio+	TiRobot™ (TI NAVI Medical Technologies Co., Ltd., Beijing, China)
Zhai2022 [25]	cohort study	ARCOⅡ	China	7/8	42	15/15	20months	Bone grafting was performed without specifying the material used.	Drilling into the necrotic lesion area and removing the cylindrical bone core	Unclear	The AIOOR System jointly developed by Inner Mongolia Medical University and Shanghai Zhuoxin Medical Technology Co., Ltd.
Li2023 [27]	cohort study	ARCOⅡ	China	36/14	47.52	50/50	28.53months	“Biolu” Ceramic Bone	Drilling into the necrotic lesion area and removing the cylindrical bone core	Osteoconductive	TiRobot™ (TI NAVI Medical Technologies Co., Ltd., Beijing, China)
Tian2023 [29]	cohort study	ARCOⅡ	China	12/7	41.4	19/30	14.6months	Allogeneic bone granules	Drilling into the necrotic lesion area and removing the cylindrical bone core	Osteoconductive	“Zhiwei Tianyan” Surgical Guidance and Feedback System (Hangzhou Santan Medical Technology Co., Ltd., China)
Ma2023 [28]	cohort study	ARCOⅡ	China	9/6	47.7	15/15	6months	Synthetic Bone Rod, composed of nano-hydroxyapatite /polyamide composite	Drilling into the necrotic lesion area and removing the cylindrical bone core	Osteoconductive	“Zhiwei Tianyan” Surgical Guidance and Feedback System (Hangzhou Santan Medical Technology Co., Ltd., China)
Cao2024 [30]	cohort study	Ficat I and Ficat II	China	19/4	47.04	23/23	12months	Artificial Fibula (Hangzhou Hongli Biomedical Technology Co., Ltd.)	Drilling into the necrotic lesion area and removing the cylindrical bone core	Osteoconductive	TiRobot™ (TI NAVI Medical Technologies Co., Ltd., Beijing, China)
Zhang2025 [31]	randomized controlled trial	ARCOⅡ	China	13/5	38.6	18/25	17.3months	Autologous bone granules	Drilling into the necrotic lesion area and removing the cylindrical bone core	Bio+	TiRobot™ (TI NAVI Medical Technologies Co., Ltd., Beijing, China)
Li2025 [1, 32]	cohort study	ARCOⅡ	China	26/12	34.6	38/38	24months	Autogenous bone paste mixed with bone marrow aspirate, and compact/multi-porous ceramic particles loaded onto a bioceramic rod.	Drilling into the necrotic lesion area and removing the cylindrical bone core	Bio+	NA

No.patients /No.hips(Number of patients / Number of hips)

TiRobot™ Orthopedic Robot (TI NAVI Medical Technologies Co., Ltd., Beijing, China)-Intraoperative C-arm fluoroscopy

GD-2000 Orthopedic Surgical Robot Navigation and Positioning System (TINAVI Medical Technologies Co., Ltd., Beijing, China)-Intraoperative C-arm fluoroscopy

Zhiwei Tianyan Surgical Navigation and Feedback System (Santan Medical Technology Co., Ltd., Hangzhou, China)-Preoperative CT + intraoperative C-arm navigation

AIOOR System (jointly developed by Inner Mongolia Medical University and Shanghai Zhuoxin Medical Technology Co., Ltd.)-Intraoperative C-arm fluoroscopy

Bio+: Bio+ refers to bone grafts that not only provide structural support and a scaffold but also contain biologically active components to promote bone regeneration

Osteoconductive: Osteoconductive means that the material provides support and acts as a scaffold to allow surrounding bone tissue to grow into it

Unclear: Unclear indicates that the relevant information was not reported or unclear

Methodological quality assessment showed that the 2 case series performed well in most appraisal items but had unclear reporting on the continuity and completeness of participant inclusion and study setting information. Among the 9 cohort studies, 1 scored 6 points, 3 scored 7 points, 4 scored 8 points, and 1 scored 9 points, indicating an overall moderate-to-high quality. The 1 RCT was rated as low risk for missing outcome data and had some concerns for the randomization process, deviations from intended interventions, outcome measurement, and selective reporting, resulting in an overall judgment of “some concerns” for risk of bias. The Cohen’s kappa coefficient indicated good inter-rater agreement. Detailed quality assessment results are presented in Supplementary Material 3.

### Meta-analysis

#### Femoral head collapse rate

A total of 11 studies reported the postoperative femoral head collapse rate [12, 13, 23–27, 29–32]. Meta-analysis showed that the pooled femoral head collapse rate in patients undergoing RACD was 9% (95% CI: 7%–13%), with low between-study heterogeneity (I² = 1.3%).

**Fig. 2:**
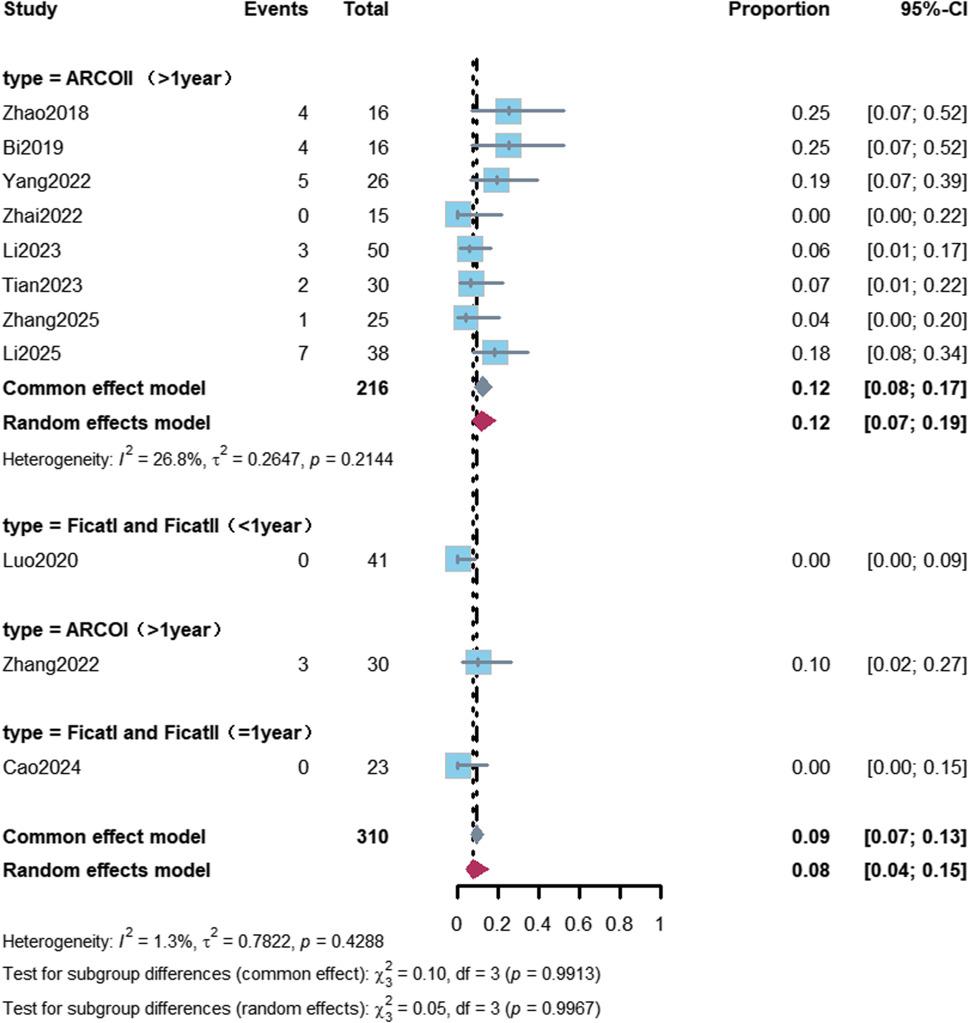
Forest plot of femoral head collapse rate stratified by follow-up duration. This figure primarily displays subgroup results stratified by stage and follow-up duration. In the subgroup of patients with ARCO stage II and follow-up >1 year (7 studies, 216 hips), the femoral head collapse rate was 12% (95% CI: 8%–17%), with low heterogeneity (I²=26.8%)

**Fig. 3:**
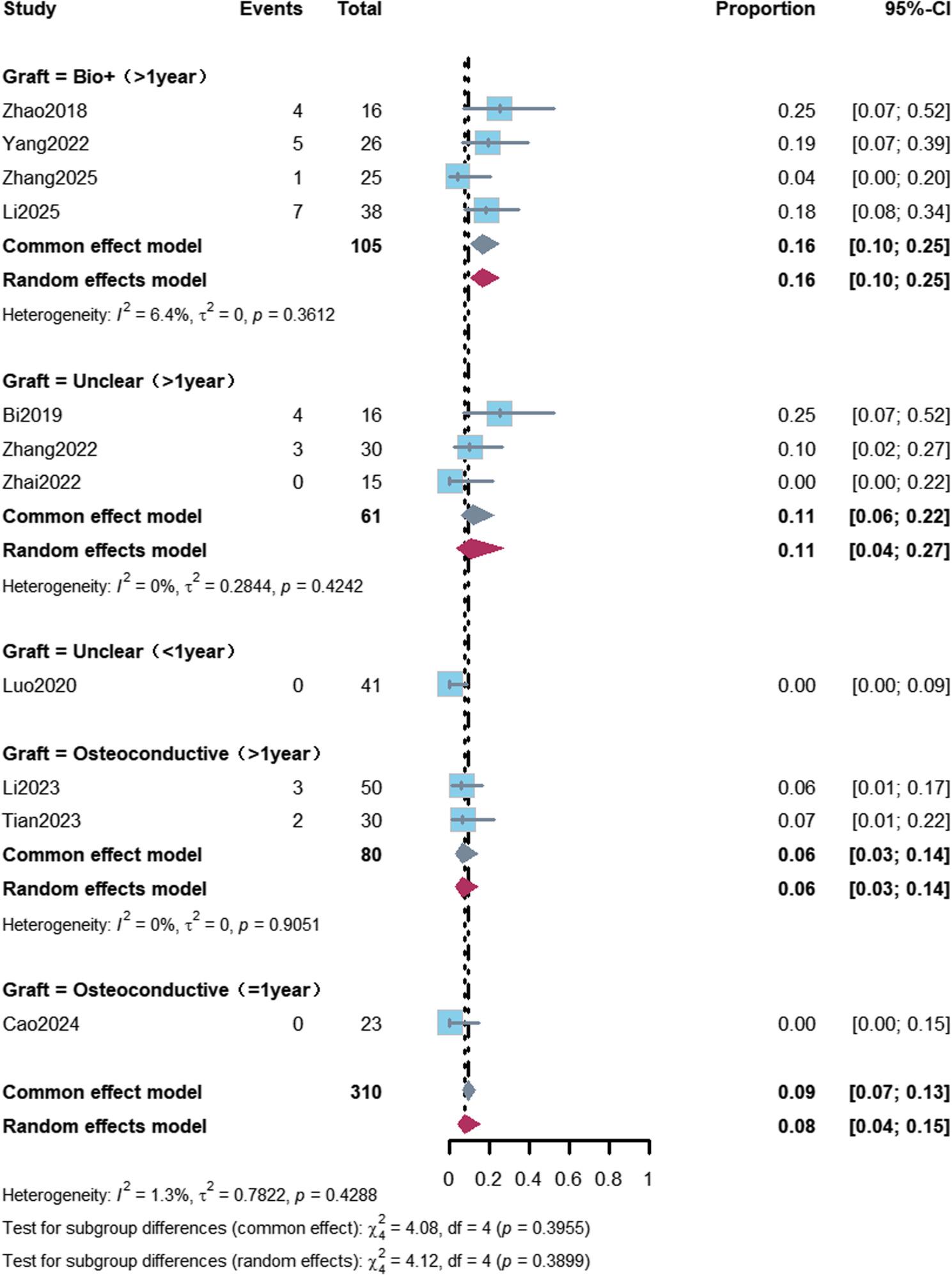
Forest plot of femoral head collapse rate stratified by graft type. Subgroup analysis of femoral head collapse rate stratified by graft type and follow-up duration. The collapse rate was 16% (95% CI: 10%–25%) in the “biological enhancement” (Bio+) subgroup and 6% (95% CI: 3%–14%) in the osteoconductive graft subgroup, both with follow-up >1 year. No significant difference was observed among subgroups (*P*=0.3899)

Subgroup analyses revealed that the femoral head collapse rate was 12% (95% CI: 8%–17%) in ARCO Stage II patients with a follow-up duration of more than 1 year, and 6% (95% CI: 3%–14%) in patients with a follow-up duration of more than 1 year who received osteoconductive bone graft materials (Figs. 2 and 3; Table 2).

**Table 2:** Results of the meta-analysis for each outcome measure

Variables	No.of study	I ^2^ (%)	ES (95%CI)	Egger	ES (95%CI)(adjusted)
Femoral Head Collapse Rate	11	1.3	0.09 (0.07, 0.13)	0.0077	0.15 (0.10, 0.22)
HHS(points)	12	96.7	17.38 (14.40–20.37)	0.0183	15.01 (10.74, 19.28)
VAS(points)	9	97.7	2.75 (2.01–3.50)	0.0378	2.28 (1.42, 3.15)

Prediction intervals before the trimming-and-filling method: HSS: 5.55–29.22, VAS: 0.03–5.47

Prediction intervals after the trimming-and-filling method: HSS: -1.24–30.42, VAS: -1.10–5.66

*HHS* Harris hip score, *VAS* Visual analog scale

#### Harris hip score

A total of 12 studies reported HHS data [12, 13, 23–32]. With a correlation coefficient of 0.5, meta-analysis showed a mean postoperative increase in HHS of 17.38 points compared with baseline (95% CI: 14.40–20.37), with high between-study heterogeneity (I² = 96.7%); the prediction interval was 5.55–29.22 points.

**Fig. 4:**
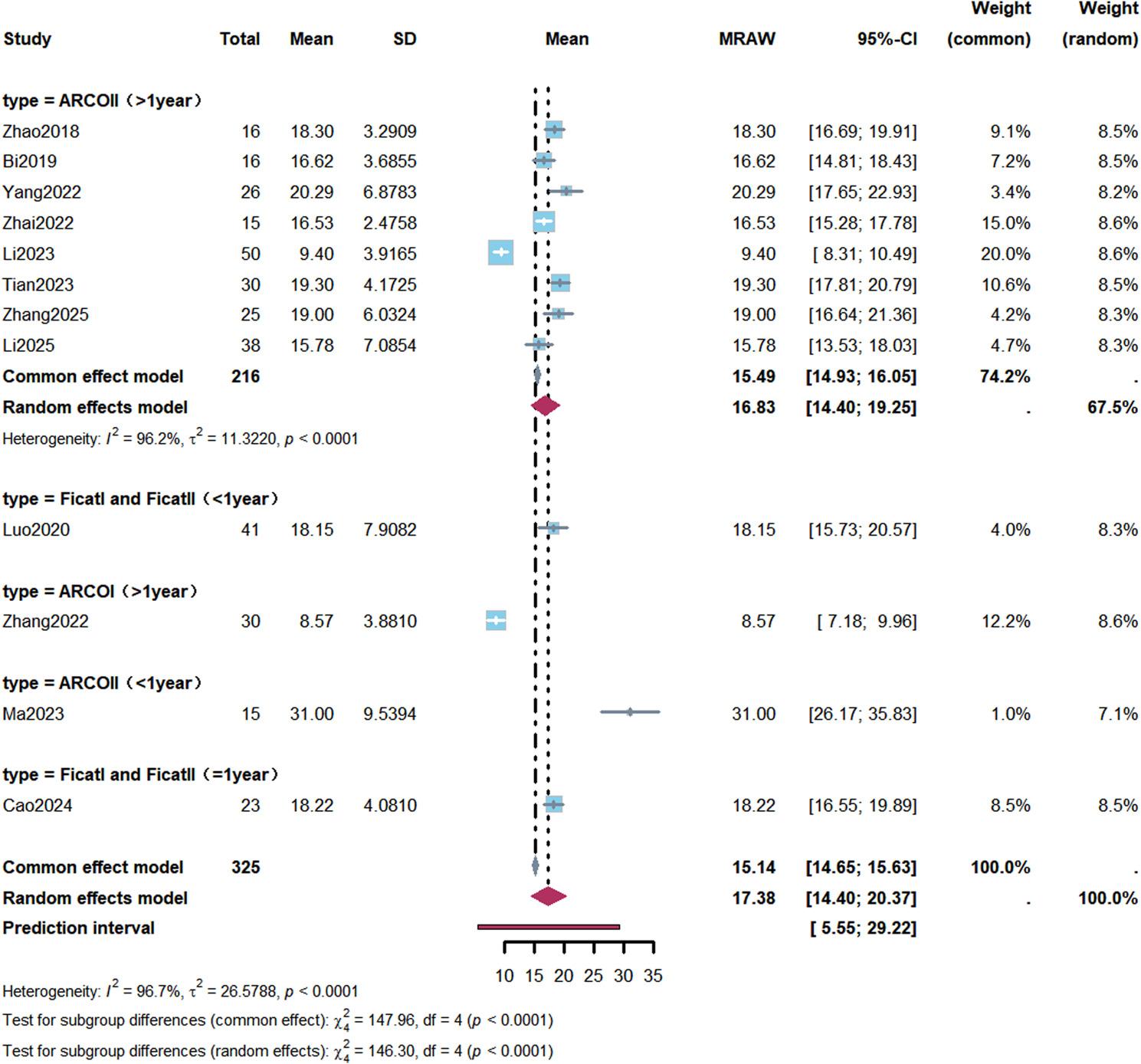
Forest plot of subgroup analysis for Harris Hip Score improvement after robot-assisted core decompression. A total of 12 studies reported Harris Hip Score (HHS) improvement. Subgroup analysis was performed according to osteonecrosis stage and follow-up duration. In patients with ARCO stage II and follow-up >1 year (8 studies, 216 patients), the mean HHS improvement was 16.83 points (95% CI: 14.40–19.25), with high heterogeneity (I²=96.2%). The pooled overall HHS improvement was 17.38 points (95% CI: 14.40–20.37), with a prediction interval of 5.55–29.22 points

**Fig. 5:**
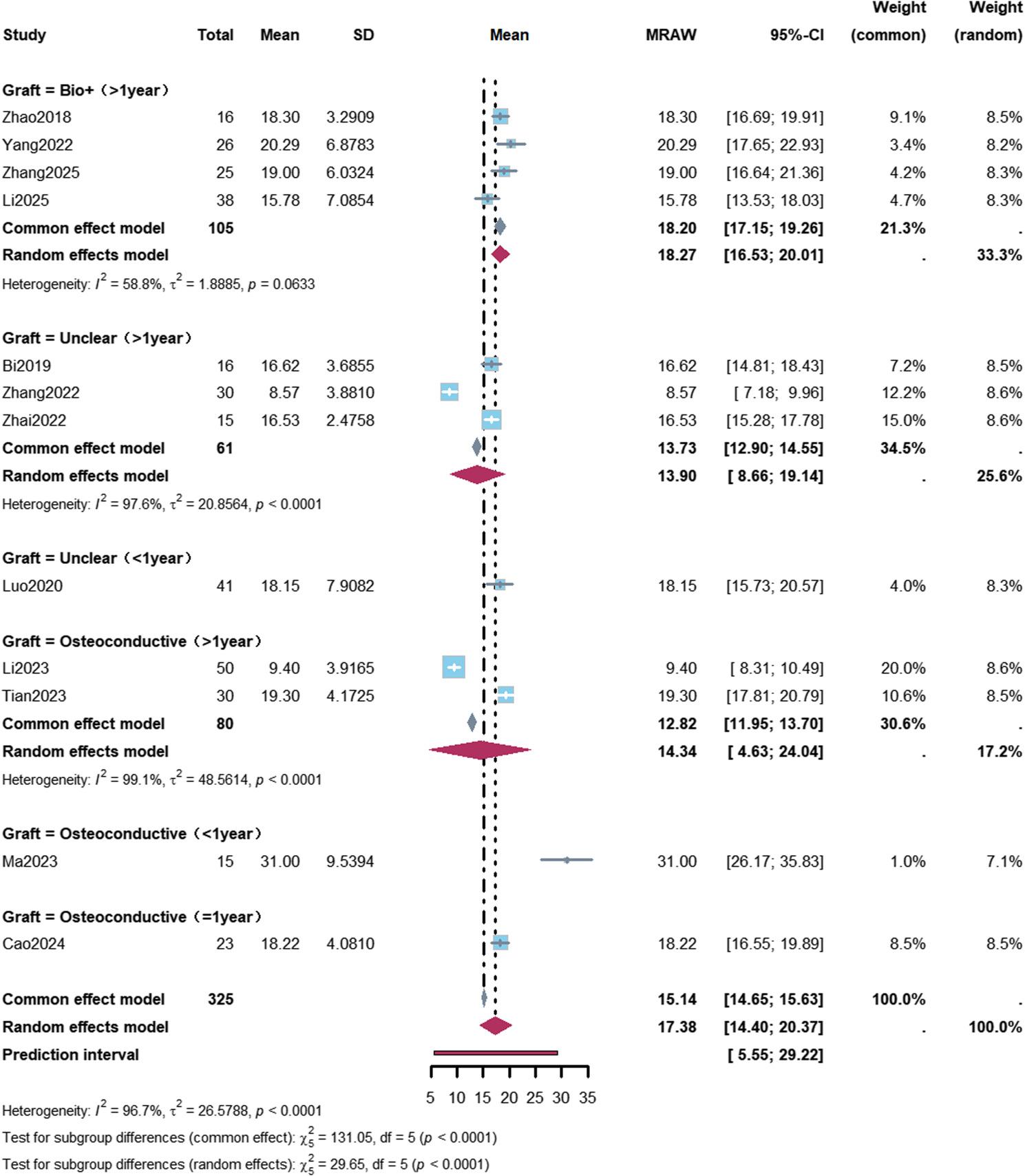
Forest plot of Harris Hip Score improvement stratified by graft type. Subgroup analysis of Harris Hip Score (HHS) improvement stratified by graft type and follow-up duration. In the “biological enhancement” (Bio+) subgroup with follow-up >1 year (4 studies, 105 patients), the mean HHS improvement was 18.27 points (95% CI: 16.53–20.01); in the osteoconductive graft subgroup (2 studies, 80 patients), the mean HHS improvement was 14.34 points (95% CI: 4.63–24.04); in the unclear graft type subgroup (3 studies, 61 patients), the mean HHS improvement was 13.90 points (95% CI: 8.66–19.14). Differences among subgroups were statistically significant (*P*<0.0001)

Subgroup analyses demonstrated a mean HHS improvement of 16.83 points (95% CI: 14.40–19.25) in ARCO Stage II patients with a follow-up duration of more than 1 year. A greater HHS improvement was observed in patients with a follow-up duration of more than 1 year who received bioactive bone graft materials, with a mean increase of 18.27 points (95% CI: 16.53–20.01) (Figs. 4 and 5; Table 2).

Sensitivity analyses with correlation coefficients of 0.25 and 0.75 showed mean postoperative HHS increases of 17.31 points (95% CI: 14.38–20.24) and 17.46 points (95% CI: 14.41–20.51), respectively. Detailed results are presented in Supplementary Material 4.

#### Visual analog scale score

A total of 9 studies reported VAS pain scores [13, 23–29, 31]. With a correlation coefficient of 0.5, meta-analysis showed a mean postoperative reduction in VAS score of 2.75 points compared with baseline (95% CI: 2.01–3.50), with high between-study heterogeneity (I² = 98.7%); the prediction interval was 0.03–5.47 points.

**Fig. 6:**
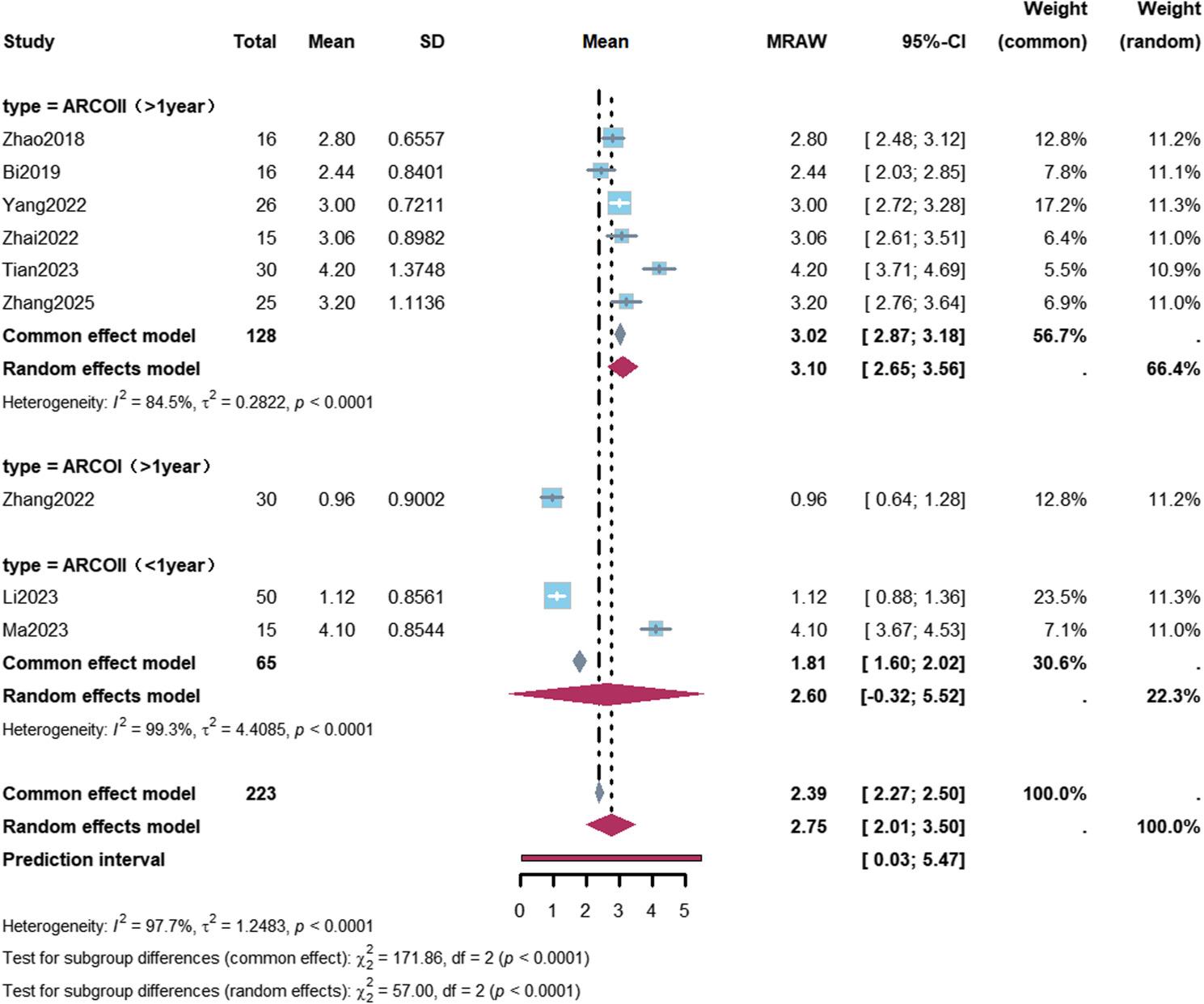
Forest plot of subgroup analysis for Visual Analogue Scale improvement after robot-assisted core decompression. A total of 9 studies reported Visual Analogue Scale (VAS) improvement. Subgroup analysis was performed according to osteonecrosis stage and follow-up duration. In patients with ARCO stage II and follow-up >1 year (6 studies, 128 patients), the mean VAS reduction was 3.10 points (95% CI: 2.65–3.56), with high heterogeneity (I²=84.5%). The pooled overall VAS improvement was 2.75 points (95% CI: 2.01–3.50), with a prediction interval of 0.03–5.47 points

**Fig. 7:**
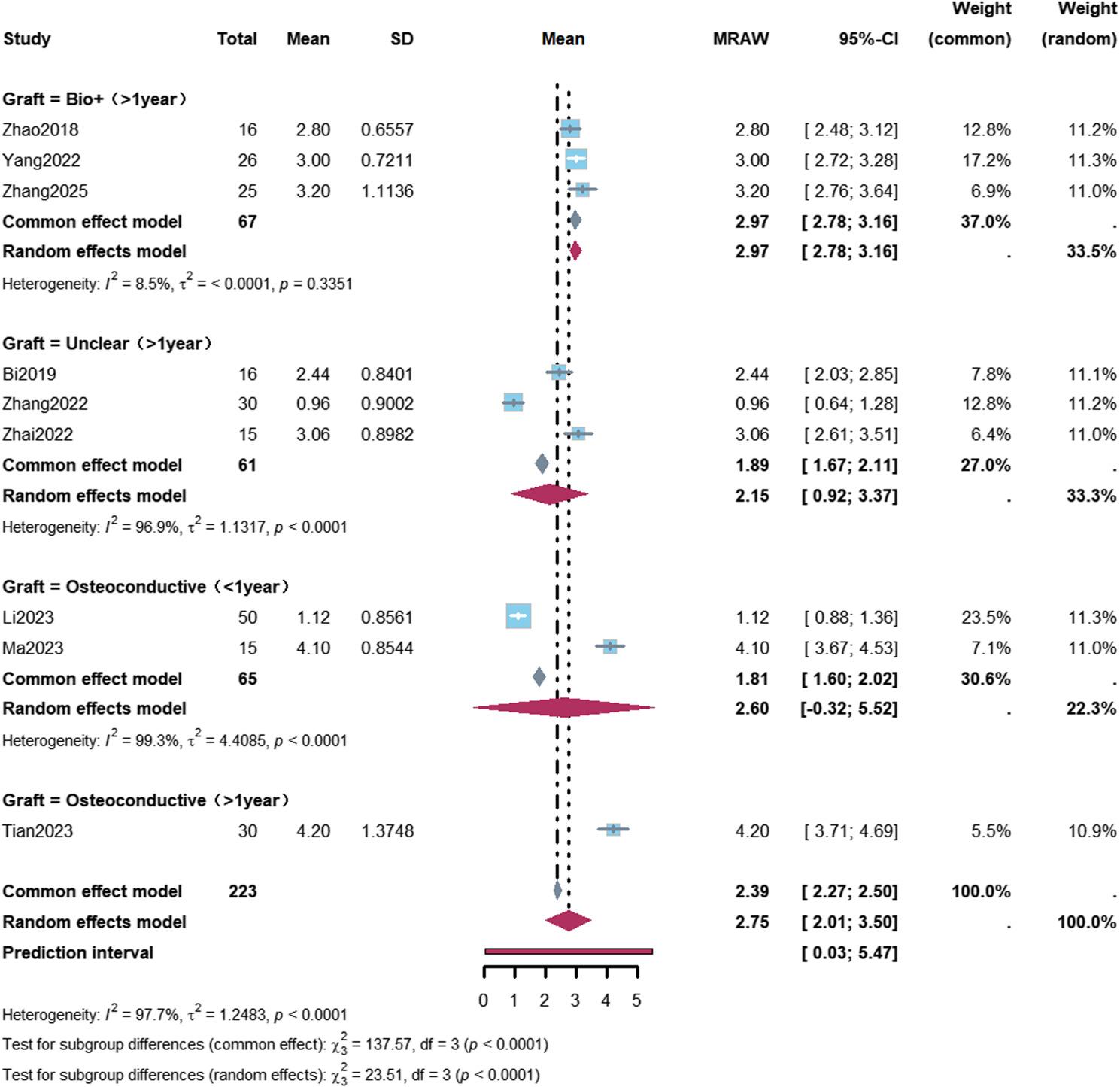
Forest plot of Visual Analogue Scale improvement stratified by graft type. Subgroup analysis of Visual Analogue Scale (VAS) improvement stratified by graft type and follow-up duration. In the “biological enhancement” (Bio+) subgroup with follow-up >1 year (3 studies, 67 patients), the mean VAS reduction was 2.97 points (95% CI: 2.78–3.16), with low heterogeneity (I²=8.5%)

Subgroup analyses revealed a mean VAS score reduction of 3.10 points (95% CI: 2.65–3.56) in ARCO Stage II patients with a follow-up duration of more than 1 year. A greater VAS improvement was observed in patients with a follow-up duration of more than 1 year who received bioactive bone graft materials, with a mean reduction of 2.97 points (95% CI: 2.78–3.16) (Figs. 6 and 7; Table 2).

Sensitivity analyses with correlation coefficients of 0.25 and 0.75 showed mean postoperative VAS reductions of 2.75 points (95% CI: 2.01–3.50) and 2.76 points (95% CI: 2.02–3.50), respectively. Detailed results are presented in Supplementary Material 4.

#### Narrative synthesis of surgery-related complications

A total of 7 studies involving 228 patients reported surgery-related complications [12, 25, 27, 29–32]. Among these, 2 studies included Ficat Stage I and II patients with mean follow-up durations of 6 months and 1 year, respectively; the remaining 5 studies included ARCO Stage II patients with a follow-up duration of more than 1 year. No surgery-related complications (e.g., infection, vascular injury, nerve injury) were reported in any of the included series during the observation period.

### Exploratory analysis

To provide a clinical reference for femoral head collapse following RACD, our supplementary analysis based on historical data showed that the femoral head collapse rate after conventional MCD was approximately 13% (17% after adjustment). The head-to-head comparison was as follows: OR 0.57, 95% CI 0.32–1.01, *P* = 0.054 (see Supplementary Material 5).

### Meta-regression

Given the significant heterogeneity in HHS and VAS improvement, separate meta-regression analyses were conducted to explore potential sources of heterogeneity.

**Fig. 8:**
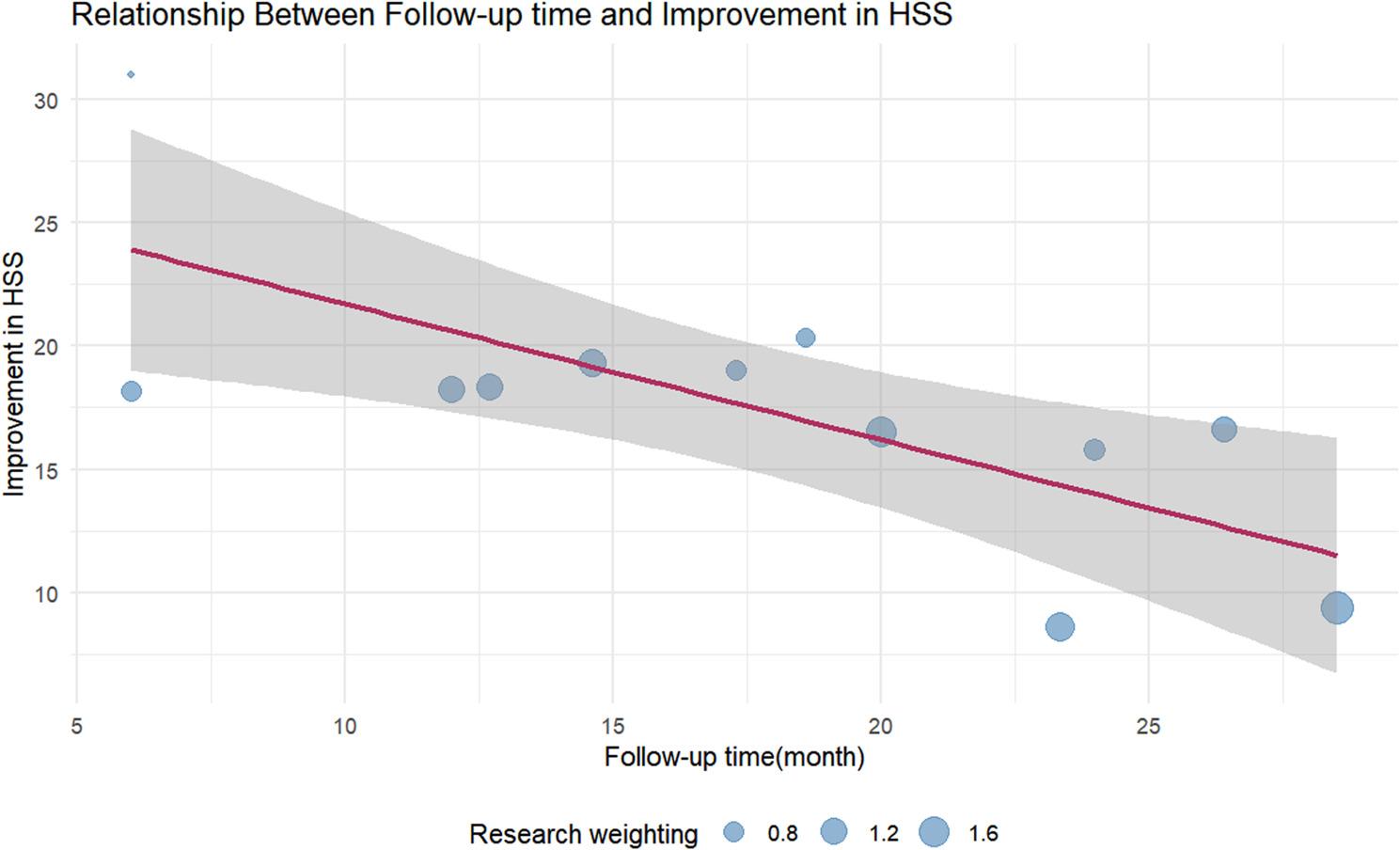
Meta-regression of follow-up duration and Harris Hip Score (HHS) improvement (weighted scatter plot). The improvement in HHS gradually decreased with longer follow-up

For HHS improvement, a statistically significant negative correlation was observed with mean follow-up duration (months) (regression coefficient β = −0.52, *P* = 0.001). The relevant weighted scatter plot is shown in Fig. 8. The model explained 51.73% of the between-study heterogeneity (*R²* = 51.73%), indicating that each additional month of follow-up was associated with a mean 0.52-point reduction in HHS improvement. No significant associations were found with other covariates (mean age, bone graft material type, ONFH stage).

**Fig. 9:**
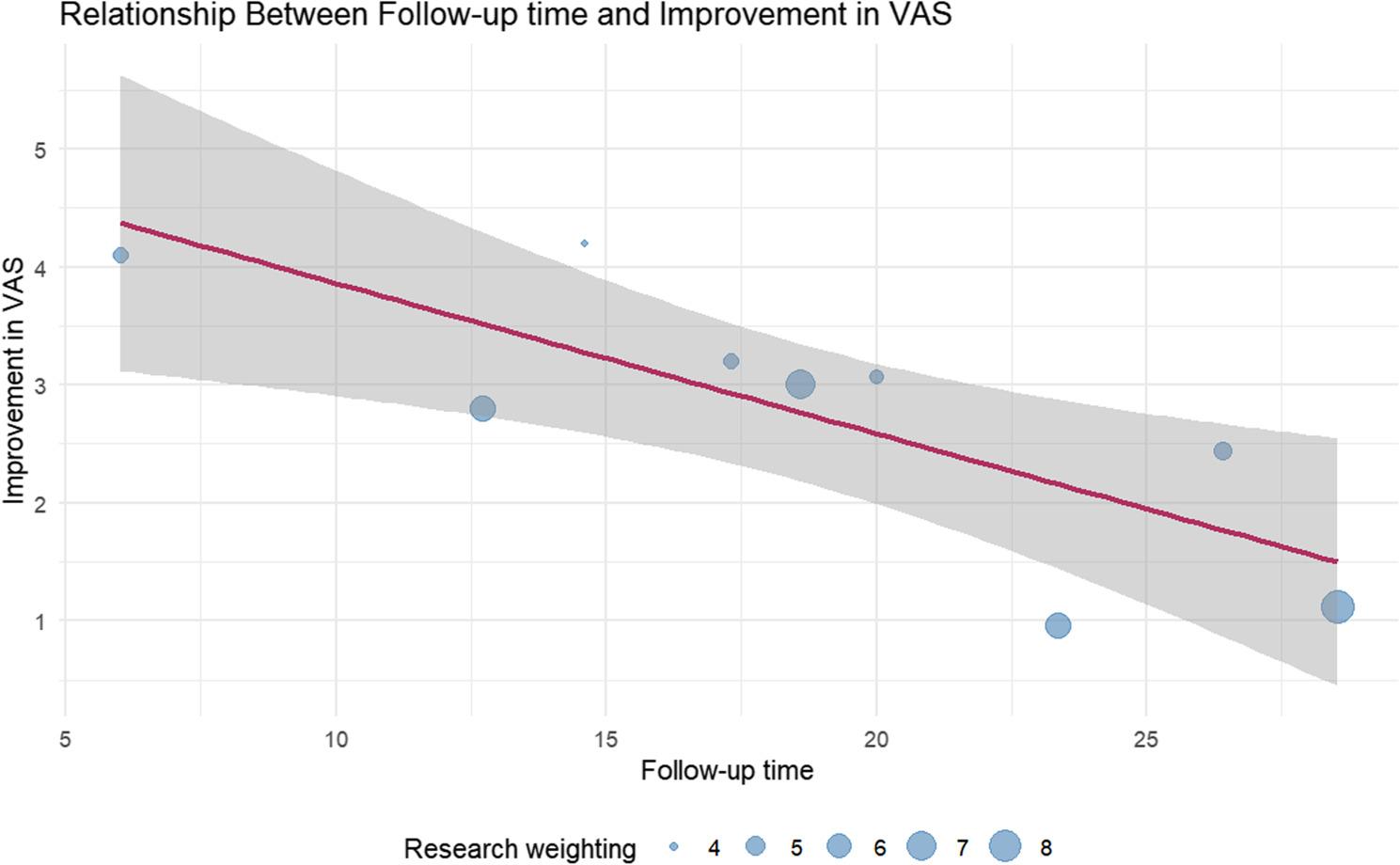
Meta-regression of follow-up duration and Visual Analogue Scale (VAS) improvement (weighted scatter plot). The improvement in VAS scores gradually decreased with longer follow-up

**Fig. 10:**
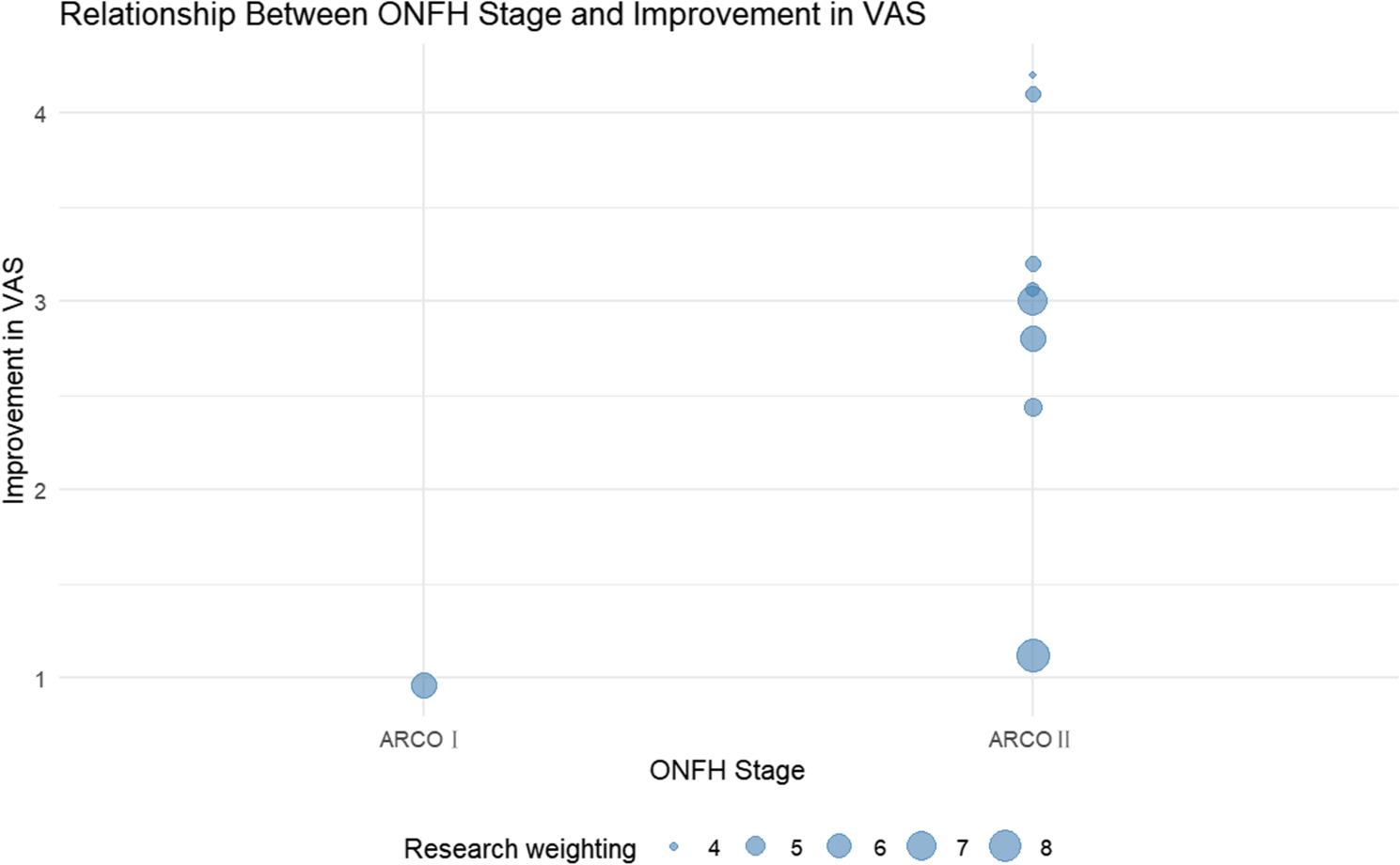
Meta-regression of osteonecrosis stage and Visual Analogue Scale improvement (weighted scatter plot). Compared with patients at ARCO stage I, the mean improvement in VAS scores was higher in those at ARCO stage II

For VAS score reduction, statistically significant associations were observed with ONFH ARCO stage (β = 2.02, *P* = 0.0004, *R*² = 26.79%) and mean follow-up duration (months) (β = −0.13, *P* = 0.0006, *R*² = 59.30%). The relevant weighted scatter plot is shown in Figs. 9 and 10. Specifically, ARCO Stage II patients had a mean VAS improvement 2.02 points greater than ARCO Stage I patients, and each additional month of follow-up was associated with a mean 0.13-point reduction in VAS improvement. A multivariate meta-regression model incorporating mean follow-up duration and ONFH ARCO stage explained 74.89% of the between-study heterogeneity (*R*² = 74.89%).

### Sensitivity analysis

Leave-one-out sensitivity analysis was performed for all outcome measures, and the pooled effect sizes remained stable after the iterative exclusion of any single study, confirming the robustness of the meta-analysis results. Detailed results are presented in Supplementary Material 6.

### Publication bias

Egger’s test indicated potential publication bias for the femoral head collapse rate (*P* = 0.0077). After imputing 4 potentially missing studies using the trim-and-fill method, the adjusted pooled femoral head collapse rate was 15% (95% CI: 10%–22%).

Potential publication bias was also observed for HHS improvement (*P* = 0.0183). After imputing 4 potentially missing studies using the trim-and-fill method, the adjusted mean HHS improvement was 14.59 points (95% CI: 11.01–18.17).

For VAS score reduction, Egger’s test detected potential publication bias (*P* = 0.0378). After imputing 2 potentially missing studies using the trim-and-fill method, the adjusted mean VAS reduction was 2.28 points (95% CI: 1.42–3.15). Detailed results are presented in Supplementary Material 7.

### According to the evidence level assessment based on the Oxford 2011 levels of evidence

Referring to the relevant content of The Oxford 2011 Levels of Evidence (Table 3), this study is a meta-analysis of single-arm studies, which meets the evaluation criteria for case series in Level 4. Therefore, the level of evidence for all outcome measures is Level 4.

**Table 3:** The Oxford 2011 Levels of Evidence

Question	Step 1 (Level 1)	Step 2 (Level 2)	Step 3 (Level 3)	Step 4 (Level 4)	Step 5 (Level 5)
How common is the problem?	Local and current random sample surveys (or censuses)	Systematic review of surveys that allow matching to local circumstances	Local non-random sample	Case-series	n/a
Does this intervention help? (Treatment Benefits)	Systematic review of randomized trials or *n*-of-1 trials	Randomized trial or observational study with dramatic effect	Non-randomized controlled cohort/follow-up study	Case-series, case-control studies, or historically controlled studies	Mechanism- based reasoning

## Discussion

This meta-analysis quantitatively evaluated the clinical efficacy of RACD for ONFH. The results showed that robotic assistance has improved precision and that has translated into improved femoral head preservation and improved function with equal or better safety standards.

This study found that the overall pooled femoral head collapse rate after RACD was 9% (95% CI: 7%–13%), and 12% (95% CI: 8%–17%) in the high-risk subgroup of ARCO Stage II patients. For clinical contextualization, indirect and direct comparisons with MCD outcomes reported in the included studies showed a higher femoral head collapse rate of approximately 13% (17% after bias adjustment) for MCD. The supplementary head-to-head comparison yielded an OR of 0.57 (95% CI: 0.32–1.01, *P* = 0.054), indicating a trend toward a reduced femoral head collapse risk with RACD that did not reach statistical significance. However, the wide 95% CI also included the possibility of no meaningful difference between the two techniques. Therefore, the inference that RACD provides superior hip preservation efficacy remains preliminary and speculative, and is not yet supported by high-quality direct comparative evidence. This favorable trend, combined with previously reported operational advantages of RACD (e.g., reduced fluoroscopy frequency, shorter operative time) [12, 13], provides contextual support for the hypothesis that improved surgical accuracy may translate into better clinical outcomes, but does not confirm this hypothesis.

Furthermore, among 222 patients from 7 included studies with limited sample sizes and follow-up durations, no complications directly related to robotic technology were reported, suggesting acceptable short-term safety of RACD. However, large-scale studies with longer follow-up durations are still required to comprehensively assess the long-term safety of this technique.

Current evidence, however, are insufficient to confirm that RACD yields superior hip preservation efficacy compared with MCD. Further direct, high-quality comparative studies are required to determine and quantify the benefits of RACD compared to MCD.

This study found a mean postoperative HHS improvement of more than 17 points after RACD, which is not only statistically significant but also exceeds the minimal clinically important difference (MCID) of 15.9 points reported for THA [33], indicating that patients experience a tangible and clinically meaningful improvement in hip function. Notably, Egger’s test suggested potential publication bias for HHS improvement (*P* = 0.0183), and the adjusted mean HHS improvement after trim-and-fill correction was 14.59 points. Although the point estimate slightly decreased, the adjusted value remains close to the MCID for THA, supporting the robustness of the core conclusion that RACD provides significant functional benefits for patients with early-stage ONFH.

Similarly, a mean postoperative VAS score reduction of 2.75 points was observed after RACD, and the adjusted mean reduction after trim-and-fill correction was 2.28 points. This value remains above the MCID for VAS improvement after THA (18.6 mm = 1.86 points) [34], confirming that the pain relief effect of RACD is clinically meaningful for patients.

Despite the significant functional and pain relief benefits of RACD, the widespread clinical adoption of this technique is hindered by its high economic cost, which mainly includes two aspects. First, the acquisition cost of robotic surgical systems is extremely high (usually millions of Chinese Yuan), and this substantial upfront capital investment can only achieve cost-effectiveness after long-term amortization over a large number of surgical cases. Second, the deployment and operation of robotic surgical systems often require corresponding operating room renovations and equipment integration (e.g., spatial layout adjustments for the robotic arm, coordination with intraoperative imaging devices such as C-arm fluoroscopy machines), which generate additional one-time capital expenditures and further increase the total initial investment.

However, the high precision inherent to RACD may bring additional prognostic benefits for patients with ONFH. Conventional MCD relies on the surgeon’s clinical experience and intraoperative fluoroscopy, which carries a certain risk of procedural error that may lead to deviated decompression tracts and severe surgical complications such as femoral neck fracture [35], thereby indirectly increasing the overall treatment cost.

From a socioeconomic perspective, the potential value of robot-assisted core decompression lies in the reduction of indirect costs. Patients with ONFH are mostly of working age. In theory, robotic surgery can delay femoral head collapse and promote recovery through precise manipulation, allowing patients to return to work earlier, thereby reducing productivity losses and generating socioeconomic benefits. However, this assumption lacks high-quality direct evidence and urgently requires further research for validation.

Core decompression itself is an effective intervention for early-stage ONFH, with its core mechanism lying in reducing intraosseous pressure and promoting revascularization. In clinical practice, “biological augmentation” (e.g., combined with autologous bone or stem cell transplantation to enhance osteogenesis) or “mechanical augmentation” (e.g., combined with structural bone grafting for mechanical support) is often added to optimize the therapeutic effect [36]. Recently, Mange et al. [37] reported arthroscopically assisted core decompression, which allows direct visualization of the hip joint during drilling and decompression. This technique can not only treat ONFH itself but also simultaneously diagnose and manage concomitant intra-articular pathologies (e.g., labral injury, synovitis, joint effusion, femoroacetabular impingement) that are important contributors to hip pain and dysfunction.

As a precision-enabled surgical tool, robotic technology systematically optimizes the established core decompression technique. Through three-dimensional precise surgical planning and stable procedural execution, RACD transforms core decompression from an experience-dependent technique into a standardized and reproducible one. By reducing intraoperative fluoroscopy exposure, accurately resecting necrotic lesions, and minimizing iatrogenic injuries such as femoral neck fracture, RACD significantly improves surgical safety. Additionally, the standardized workflow of RACD lowers the learning curve for orthopedic surgeons, facilitating the wider dissemination of this high-quality surgical technique.

Therefore, the core clinical value of robotic technology in ONFH treatment is that it is expected to enable a proven and effective “good technique” (core decompression) to achieve its theoretically optimal therapeutic outcomes more safely and consistently in a broader patient population.

However, the reporting methods used in existing studies are highly inconsistent, which severely hinders our in-depth exploration of the genuine advantages of robotic technology and evidence synthesis. Lesion size should be reported consistently, and outcomes should be stratified by lesion size (e.g., the respective collapse rates and THA rates for necrotic areas < 15%, 15%–30%, and > 30%, etc.). This will help us identify the optimal indications for robotic technology.

Furthermore, the advantages of robotic assistance in improving precision may only be fully revealed through long-term follow-up studies. This is primarily due to the pathological characteristics of ONFH itself: it is a slowly progressive disease. The short-term benefits conferred by robotic technology—such as more precise debridement of necrotic lesions—may not immediately translate into a significantly higher hip-preservation success rate than conventional surgery within the short follow-up period of 1–2 years, but instead require longer-term follow-up and observation.This is because short-term success depends largely on whether the surgery resolves the immediate problems (e.g., intraosseous hypertension), whereas long-term success relies on whether the long-term biological and mechanical environment created by the procedure for the femoral head is sufficiently stable to resist the natural progression of the disease.

During the review of relevant studies, we found that the femoral head collapse rate in ARCO stage Ⅱc patients after core decompression was much higher than that in other stages. For example, in the study by Tian et al., 11 patients in the MCD group experienced femoral head collapse, among whom 7 were preoperatively classified as ARCO stage Ⅱc [29]. In the RACD group, 2 patients had femoral head collapse, both of whom were preoperatively assessed as ARCO stage Ⅱc [29].

Therefore, we hypothesize that head-to-head comparisons focusing on patients with ARCO stages IIa and IIb may reveal greater differences during long-term follow-up. Future studies could prioritize this aspect and conduct relevant research to verify our hypothesis.

Despite the favorable trends in the key outcomes of RACD observed in this study, the findings must be interpreted with caution due to several important limitations. First, the included studies were mainly observational studies (case series, cohort studies), resulting in an overall low level of evidence. Although the pooled HHS and VAS results showed high between-study heterogeneity, meta-regression identified follow-up duration as a key source of heterogeneity, which improved the interpretability of the results.

Second, Egger’s test indicated potential publication bias for femoral head collapse rate and HHS improvement; after trim-and-fill correction, the femoral head collapse rate increased from 9% to 15%, and the mean HHS improvement decreased from 17.38 points to 14.59 points. This suggests that although the positive findings of RACD remain valid, the effect sizes may have been overestimated in the published literature. Furthermore, Egger’s test and the trim-and-fill method may have inherent instability, and the corrected results should therefore be interpreted cautiously.

Third, all included studies were conducted in China, which limits the generalizability of the findings to other populations and regions. In addition, surgical experience (including patient selection, individualized surgical planning, and intraoperative complication management) may influence long-term outcomes such as femoral head collapse after surgeons overcome the initial learning curve of RACD through systematic training and accumulated case volume. Therefore, further studies from other countries and regions are needed to validate the results of this study, and future studies should detail the surgical experience of the operating surgeons and teams to evaluate the impact of surgical experience on long-term ONFH outcomes.

Fourth, very few included studies reported details on THA conversion after femoral head collapse, which prevented a comprehensive assessment of the THA conversion rate after RACD. Future studies should systematically report THA-related outcomes to enable a more comprehensive evaluation of the long-term clinical efficacy of RACD.

Furthermore, the etiology and lesion size of ONFH were not systematically reported in the included studies, which prevented us from further evaluating the efficacy of robot‑assisted core decompression in patients with different etiologies and lesion sizes. According to previous studies, the most ideal lesions treated with core decompression are precollapse and small‑volume necrotic lesions (less than 15% of the femoral head involved or Kerboul angle < 200°) [38–41]. Future clinical studies may consider reporting these data, which can provide more information for subsequent meta‑analyses.

Finally, the mean follow-up duration in most included studies was short-to-medium term (1–3 years). Since ONFH is a progressive chronic disease, longer follow-up durations (e.g., 5 and 10 years) are essential for evaluating definitive hip preservation success and the delay of THA.

Based on the current evidence, RACD can be considered a promising minimally invasive intervention for patients with early-stage ONFH (especially ARCO Stages I–II). Future research should prioritize large-sample, long-term follow-up, multicenter RCTs with head-to-head comparisons between RACD and MCD, and the results of this study can provide important parameter support for sample size calculation in such future studies.

Furthermore, considering that demonstrating the advantages of robotic assistance requires the implementation of long-term randomized controlled trials, which entail substantial human and material resources, the eventual benefit of robotic assistance may still be minimal [42]. Therefore, identifying the risk factors for femoral head collapse after core decompression and applying robot-assisted technology selectively to high-risk populations may be more cost-effective for patients.

## Conclusions

Available evidence supports the effectiveness of robot-assisted core decompression in the treatment of early-stage ONFH. RACD yields functional improvement and pain relief in patients with ONFH, with acceptable short-to-medium-term safety. Current evidence, however, are insufficient to confirm that RACD yields superior hip preservation efficacy compared with MCD. Further direct, high-quality comparative studies are required to determine and quantify the benefits of RACD compared to MCD.

## Supplementary Information

Supplementary Material 1: Material 1: Systematic Literature Search Strategy. Material 2: Details of duplicate study identification. Material 3: Quality Assessment of Included Studies. Material 4：Analysis results of different correlation coefficients for HSS and VAS (0.25 and 0.75). Material 5: Supplementary Analysis on Manual core Decompression. Material 6: Sensitivity Analysis Results. Material 7: The results of the trim-and-fill method for the femoral head collapse rate, HHS and VAS.

Supplementary Material 2.

## Data Availability

All data generated or analysed during this study are included in this published article [and its supplementary information files].

## Abbreviations

ONFH: Osteonecrosis of the femoral head
RACD: Robot-assisted core decompression
MCD: Manual core decompression
RCT: Randomized controlled trial
CI: Confidence interval
MD: Mean difference
HHS: Harris Hip Score
VAS: Visual Analogue Scale
THA: Total hip arthroplasty
MCID: Minimal clinically important difference
ARCO: Association Research Circulation Osseous
NOS: Newcastle–Ottawa Scale
RoB 2: Risk of Bias 2
GLMM: Generalized linear mixed model
REML: Restricted maximum likelihood estimation
OR: Odds ratio
