# Supplementary material for: Efficacy and safety of robot-assisted core decompression for osteonecrosis of the femoral head: a meta-analysis of single-arm studies
Source: BMC Musculoskelet Disord. 2026 May 6;27:387. doi: 10.1186/s12891-026-09777-y (PMC13151216; doi:10.1186/s12891-026-09777-y)
Supplement: Supplementary file 1 — Supplementary Material 1: Material 1: Systematic Literature Search Strategy. Material 2: Details of duplicate study identification. Material 3: Quality Assessment of Included Studies. Material 4：Analysis results of different correlation coefficients for HSS and VAS (0.25 and 0.75). Material 5: Supplementary Analysis on Manual core Decompression. Material 6: Sensitivity Analysis Results. Material 7: The results of the trim-and-fill method for the femoral head collapse rate, HHS and VAS. [file 12891_2026_9777_MOESM1_ESM.docx]

**Supplementary Material 1: Systematic Literature Search Strategy**

**Supplementary Material 2: Details of duplicate study identification**

**Supplementary Material 3: Quality Assessment of Included Studies**

****Supplementary Material 4：Analysis results of different correlation coefficients for HSS and VAS (0.25 and 0.75)****

****Supplementary Material 5:** Supplementary Analysis on Manual core Decompression**

**Supplementary Material 6: Sensitivity Analysis Results**

**Supplementary Material 7: The results of the trim-and-fill method for the femoral head collapse rate, HHS and VAS**

**Supplementary Material 1. Systematic Literature Search Strategy1.PubMed**

| **Search number** | **Query** | **Results** |
| --- | --- | --- |
| 1 | "Robotic Surgical Procedures"[Mesh] | 23,659 |
| 2 | ((((((((((((((((((((((((Robotic Surgical Procedures[Title/Abstract]) OR (Procedure, Robotic Surgical[Title/Abstract])) OR (Procedures, Robotic Surgical[Title/Abstract])) OR (Robotic Surgical Procedure[Title/Abstract])) OR (Surgical Procedure, Robotic[Title/Abstract])) OR (Robotic-Assisted Surgery[Title/Abstract])) OR (Robotic-Assisted Surgeries[Title/Abstract])) OR (Robotic Assisted Surgery[Title/Abstract])) OR (Surgery, Robotic-Assisted[Title/Abstract])) OR (Surgical Procedures, Robotic[Title/Abstract])) OR (Robot-Assisted Surgery[Title/Abstract])) OR (Robot-Assisted Surgeries[Title/Abstract])) OR (Robot Assisted Surgery[Title/Abstract])) OR (Surgery, Robot-Assisted[Title/Abstract])) OR (Robot Surgery[Title/Abstract])) OR (Robot Surgeries[Title/Abstract])) OR (Surgery, Robot[Title/Abstract])) OR (Robot-Enhanced Procedures[Title/Abstract])) OR (Procedure, Robot-Enhanced[Title/Abstract])) OR (Robot-Enhanced Procedure[Title/Abstract])) OR (Robot Enhanced Procedures[Title/Abstract])) OR (Robot-Enhanced Surgery[Title/Abstract])) OR (Robot-Enhanced Surgeries[Title/Abstract])) OR (Robot Enhanced Surgery[Title/Abstract])) OR (Surgery, Robot-Enhanced[Title/Abstract]) | 7,514 |
| 3 | ("Robotic Surgical Procedures"[Mesh]) OR (((((((((((((((((((((((((Robotic Surgical Procedures[Title/Abstract]) OR (Procedure, Robotic Surgical[Title/Abstract])) OR (Procedures, Robotic Surgical[Title/Abstract])) OR (Robotic Surgical Procedure[Title/Abstract])) OR (Surgical Procedure, Robotic[Title/Abstract])) OR (Robotic-Assisted Surgery[Title/Abstract])) OR (Robotic-Assisted Surgeries[Title/Abstract])) OR (Robotic Assisted Surgery[Title/Abstract])) OR (Surgery, Robotic-Assisted[Title/Abstract])) OR (Surgical Procedures, Robotic[Title/Abstract])) OR (Robot-Assisted Surgery[Title/Abstract])) OR (Robot-Assisted Surgeries[Title/Abstract])) OR (Robot Assisted Surgery[Title/Abstract])) OR (Surgery, Robot-Assisted[Title/Abstract])) OR (Robot Surgery[Title/Abstract])) OR (Robot Surgeries[Title/Abstract])) OR (Surgery, Robot[Title/Abstract])) OR (Robot-Enhanced Procedures[Title/Abstract])) OR (Procedure, Robot-Enhanced[Title/Abstract])) OR (Robot-Enhanced Procedure[Title/Abstract])) OR (Robot Enhanced Procedures[Title/Abstract])) OR (Robot-Enhanced Surgery[Title/Abstract])) OR (Robot-Enhanced Surgeries[Title/Abstract])) OR (Robot Enhanced Surgery[Title/Abstract])) OR (Surgery, Robot-Enhanced[Title/Abstract])) | 27,187 |
| 4 | "Femur Head Necrosis"[Mesh] | 9,275 |
| 5 | ((((((((((Femur Head Necrosis[Title/Abstract]) OR (Femur Head Necroses[Title/Abstract])) OR (Head Necrosis, Femur[Title/Abstract])) OR (Necrosis, Femur Head[Title/Abstract])) OR (Necrosis, Avascular, of Femur Head[Title/Abstract])) OR (Avascular Necrosis Of Femoral Head, Primary[Title/Abstract])) OR (Avascular Necrosis of Femur Head[Title/Abstract])) OR (Femoral Head, Avascular Necrosis Of[Title/Abstract])) OR (Ischemic Necrosis Of Femoral Head[Title/Abstract])) OR (Aseptic Necrosis of Femur Head[Title/Abstract])) OR (Necrosis, Aseptic, of Femur Head[Title/Abstract]) | 1,786 |
| 6 | ("Femur Head Necrosis"[Mesh]) OR (((((((((((Femur Head Necrosis[Title/Abstract]) OR (Femur Head Necroses[Title/Abstract])) OR (Head Necrosis, Femur[Title/Abstract])) OR (Necrosis, Femur Head[Title/Abstract])) OR (Necrosis, Avascular, of Femur Head[Title/Abstract])) OR (Avascular Necrosis Of Femoral Head, Primary[Title/Abstract])) OR (Avascular Necrosis of Femur Head[Title/Abstract])) OR (Femoral Head, Avascular Necrosis Of[Title/Abstract])) OR (Ischemic Necrosis Of Femoral Head[Title/Abstract])) OR (Aseptic Necrosis of Femur Head[Title/Abstract])) OR (Necrosis, Aseptic, of Femur Head[Title/Abstract])) | 9,862 |
| 7 | (("Robotic Surgical Procedures"[Mesh]) OR (((((((((((((((((((((((((Robotic Surgical Procedures[Title/Abstract]) OR (Procedure, Robotic Surgical[Title/Abstract])) OR (Procedures, Robotic Surgical[Title/Abstract])) OR (Robotic Surgical Procedure[Title/Abstract])) OR (Surgical Procedure, Robotic[Title/Abstract])) OR (Robotic-Assisted Surgery[Title/Abstract])) OR (Robotic-Assisted Surgeries[Title/Abstract])) OR (Robotic Assisted Surgery[Title/Abstract])) OR (Surgery, Robotic-Assisted[Title/Abstract])) OR (Surgical Procedures, Robotic[Title/Abstract])) OR (Robot-Assisted Surgery[Title/Abstract])) OR (Robot-Assisted Surgeries[Title/Abstract])) OR (Robot Assisted Surgery[Title/Abstract])) OR (Surgery, Robot-Assisted[Title/Abstract])) OR (Robot Surgery[Title/Abstract])) OR (Robot Surgeries[Title/Abstract])) OR (Surgery, Robot[Title/Abstract])) OR (Robot-Enhanced Procedures[Title/Abstract])) OR (Procedure, Robot-Enhanced[Title/Abstract])) OR (Robot-Enhanced Procedure[Title/Abstract])) OR (Robot Enhanced Procedures[Title/Abstract])) OR (Robot-Enhanced Surgery[Title/Abstract])) OR (Robot-Enhanced Surgeries[Title/Abstract])) OR (Robot Enhanced Surgery[Title/Abstract])) OR (Surgery, Robot-Enhanced[Title/Abstract]))) AND (("Femur Head Necrosis"[Mesh]) OR (((((((((((Femur Head Necrosis[Title/Abstract]) OR (Femur Head Necroses[Title/Abstract])) OR (Head Necrosis, Femur[Title/Abstract])) OR (Necrosis, Femur Head[Title/Abstract])) OR (Necrosis, Avascular, of Femur Head[Title/Abstract])) OR (Avascular Necrosis Of Femoral Head, Primary[Title/Abstract])) OR (Avascular Necrosis of Femur Head[Title/Abstract])) OR (Femoral Head, Avascular Necrosis Of[Title/Abstract])) OR (Ischemic Necrosis Of Femoral Head[Title/Abstract])) OR (Aseptic Necrosis of Femur Head[Title/Abstract])) OR (Necrosis, Aseptic, of Femur Head[Title/Abstract]))) | 6 |

**2.Embase**

| **Search number** | **Query** | **Results** |
| --- | --- | --- |
| 1 | 'robot assisted surgery'/exp | 46194 |
| 2 | 'robot assisted surgery':ab,ti | 2572 |
| 3 | 'procedure, robotic surgical':ab,ti | 3 |
| 4 | 'procedures, robotic surgical':ab,ti | 7 |
| 5 | 'surgical procedure, robotic':ab,ti | 17 |
| 6 | 'robotic-assisted surgery':ab,ti | 2452 |
| 7 | 'robotic-assisted surgeries':ab,ti | 134 |
| 8 | 'robotic assisted surgery':ab,ti | 2452 |
| 9 | 'surgery, robotic-assisted':ab,ti | 122 |
| 10 | 'surgical procedures, robotic':ab,ti | 9 |
| 11 | 'robot-assisted surgery':ab,ti | 2573 |
| 12 | 'robot-assisted surgeries':ab,ti | 174 |
| 13 | 'robot assisted surgery':ab,ti | 2572 |
| 14 | 'surgery, robot-assisted':ab,ti | 176 |
| 15 | 'robot surgery':ab,ti | 18786 |
| 16 | 'robot surgeries':ab,ti | 2254 |
| 17 | 'surgery, robot':ab,ti | 309 |
| 18 | 'robot-enhanced procedures':ab,ti | 0 |
| 19 | 'procedure, robot-enhanced':ab,ti | 0 |
| 20 | 'robot-enhanced procedure':ab,ti | 0 |
| 21 | 'robot-enhanced procedures':ab,ti | 0 |
| 22 | 'robot-enhanced surgery':ab,ti | 5 |
| 23 | 'robot-enhanced surgeries':ab,ti | 0 |
| 24 | 'robot enhanced surgery':ab,ti | 5 |
| 25 | 'surgery, robot-enhanced':ab,ti | 0 |
| 26 | #1 OR #2 OR #3 OR #4 OR #5 OR #6 OR #7 OR #8 OR #9 OR #10 OR #11 OR #12 OR #13 OR #14 OR #15 OR #16 OR #17 OR #18 OR #19 OR #20 OR #21 OR #22 OR #23 OR #24 OR #25 | 56858 |
| 27 | 'femur head necrosis'/exp | 8736 |
| 28 | 'femur head necrosis':ab,ti | 158 |
| 29 | 'femur head necroses':ab,ti | 11 |
| 30 | 'head necrosis, femur':ab,ti | 0 |
| 31 | 'necrosis, femur head':ab,ti | 1 |
| 32 | 'necrosis, avascular, of femur head':ab,ti | 0 |
| 33 | 'avascular necrosis of femoral head, primary':ab,ti | 0 |
| 34 | 'avascular necrosis of femur head':ab,ti | 14 |
| 35 | 'femoral head, avascular necrosis of':ab,ti | 5 |
| 36 | 'ischemic necrosis of femoral head':ab,ti | 44 |
| 37 | 'aseptic necrosis of femur head':ab,ti | 6 |
| 38 | 'necrosis, aseptic, of femur head':ab,ti | 0 |
| 39 | #27 OR #28 OR #29 OR #30 OR #31 OR #32 OR #33 OR #34 OR #35 OR #36 OR #37 OR #38 | 8797 |
| 40 | #26 AND #39 | 25 |

**3.Cochrane library**

| **Search number** | **Query** | **Results** |
| --- | --- | --- |
| 1 | MeSH descriptor: [Robotic Surgical Procedures] explode all trees | 1121 |
| 2 | (Robotic Surgical Procedures):ti,ab,kw OR (Procedure, Robotic Surgical):ti,ab,kw OR (Procedures, Robotic Surgical):ti,ab,kw OR (Robotic Surgical Procedure):ti,ab,kw OR (Surgical Procedure, Robotic):ti,ab,kw | 1904 |
| 3 | (Robotic-Assisted Surgery):ti,ab,kw OR (Robotic-Assisted Surgeries):ti,ab,kw OR (Robotic Assisted Surgery):ti,ab,kw OR (Surgery, Robotic-Assisted):ti,ab,kw OR (Surgical Procedures, Robotic):ti,ab,kw | 2718 |
| 4 | (Robot-Assisted Surgery):ti,ab,kw OR (Robot-Assisted Surgeries):ti,ab,kw OR (Robot Assisted Surgery):ti,ab,kw OR (Surgery, Robot-Assisted):ti,ab,kw OR (Robot Surgery):ti,ab,kw | 2466 |
| 5 | (Robot Surgeries):ti,ab,kw OR (Surgery, Robot):ti,ab,kw OR (Robot-Enhanced Procedures):ti,ab,kw OR (Procedure, Robot-Enhanced):ti,ab,kw OR (Robot-Enhanced Procedure):ti,ab,kw | 2470 |
| 6 | (Robot Enhanced Procedures):ti,ab,kw OR (Robot-Enhanced Surgery):ti,ab,kw OR (Robot-Enhanced Surgeries):ti,ab,kw OR (Robot Enhanced Surgery):ti,ab,kw OR (Surgery, Robot-Enhanced):ti,ab,kw | 120 |
| 7 | #1 or #2 or #3 or #4 or #5 or #6 | 199 |
| 8 | MeSH descriptor: [Femur Head Necrosis] explode all trees | 34 |
| 9 | (Femur Head Necroses):ti,ab,kw OR (Femur Head Necrosis):ti,ab,kw OR (Head Necrosis, Femur):ti,ab,kw OR (Necrosis, Femur Head):ti,ab,kw OR (Necrosis, Avascular, of Femur Head):ti,ab,kw | 295 |
| 10 | (Avascular Necrosis Of Femoral Head, Primary):ti,ab,kw OR (Avascular Necrosis of Femur Head):ti,ab,kw OR (Femoral Head, Avascular Necrosis Of):ti,ab,kw OR (Ischemic Necrosis Of Femoral Head):ti,ab,kw OR (Aseptic Necrosis of Femur Head):ti,ab,kw | 239 |
| 11 | Necrosis, Aseptic, of Femur Head | 22 |
| 12 | #8 or #9 or #10 or #11 | 459 |
| 13 | #7 and #12 | 3 |

**4.Web of science**

| **Search number** | **Query** | **Results** |
| --- | --- | --- |
| 1 | ((((((((((((((((((((((((TS=(Robotic Surgical Procedures)) OR TS=(Procedure, Robotic Surgical)) OR TS=(Procedures, Robotic Surgical)) OR TS=(Robotic Surgical Procedure)) OR TS=(Surgical Procedure, Robotic)) OR TS=(Robotic-Assisted Surgery)) OR TS=(Robotic-Assisted Surgeries)) OR TS=(Robotic Assisted Surgery)) OR TS=(Surgery, Robotic-Assisted)) OR TS=(Surgical Procedures, Robotic)) OR TS=(Robot-Assisted Surgery)) OR TS=(Robot-Assisted Surgeries)) OR TS=(Robot Assisted Surgery)) OR TS=(Surgery, Robot-Assisted)) OR TS=(Robot Surgery)) OR TS=(Robot Surgeries)) OR TS=(Surgery, Robot)) OR TS=(Robot-Enhanced Procedures)) OR TS=(Procedure, Robot-Enhanced)) OR TS=(Robot-Enhanced Procedure)) OR TS=(Robot Enhanced Procedures)) OR TS=(Robot-Enhanced Surgery)) OR TS=(Robot-Enhanced Surgeries)) OR TS=(Robot Enhanced Surgery)) OR TS=(Surgery, Robot-Enhanced) | 36519 |
| 2 | ((((((((((TS=(Femur Head Necrosis)) OR TS=(Femur Head Necroses)) OR TS=(Head Necrosis, Femur)) OR TS=(Necrosis, Femur Head)) OR TS=(Necrosis, Avascular, of Femur Head)) OR TS=(Avascular Necrosis Of Femoral Head, Primary)) OR TS=(Avascular Necrosis of Femur Head)) OR TS=(Femoral Head, Avascular Necrosis Of)) OR TS=(Ischemic Necrosis Of Femoral Head)) OR TS=(Aseptic Necrosis of Femur Head)) OR TS=(Necrosis, Aseptic, of Femur Head) | 5478 |
| 3 | #1 AND #2 | 4 |

1. CNKI：41 （主题：股骨头缺血坏死）OR（主题：股骨头坏死）OR（主题：股骨头缺血性坏死）OR（主题：股骨头无菌坏死）OR（主题：股骨头无菌性坏死）OR（主题：股骨坏死）AND（主题：机器人）


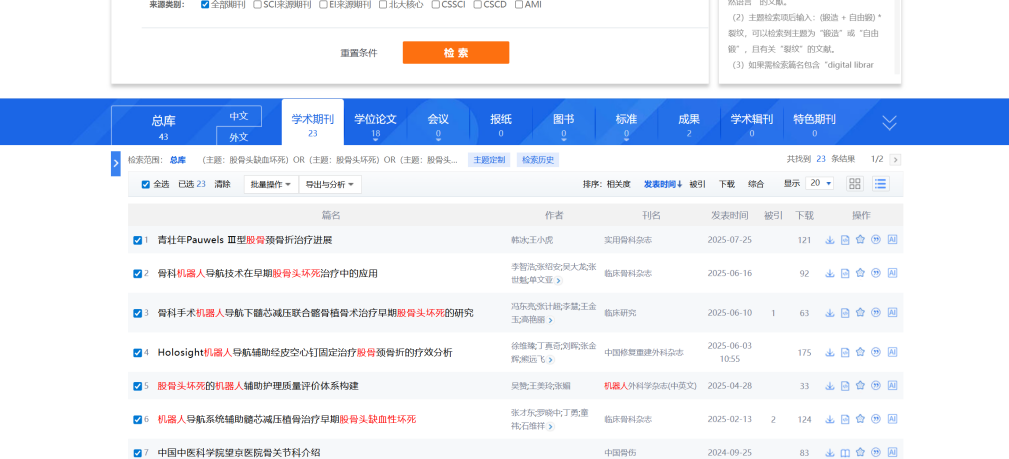


6. Wanfang：69 主题:(股骨头缺血坏死 O R股骨头坏死 OR 股骨头缺血性坏死 OR 股骨头无菌坏死 OR 股骨头无菌性坏死 OR 股骨坏死) and 主题:(机器人)


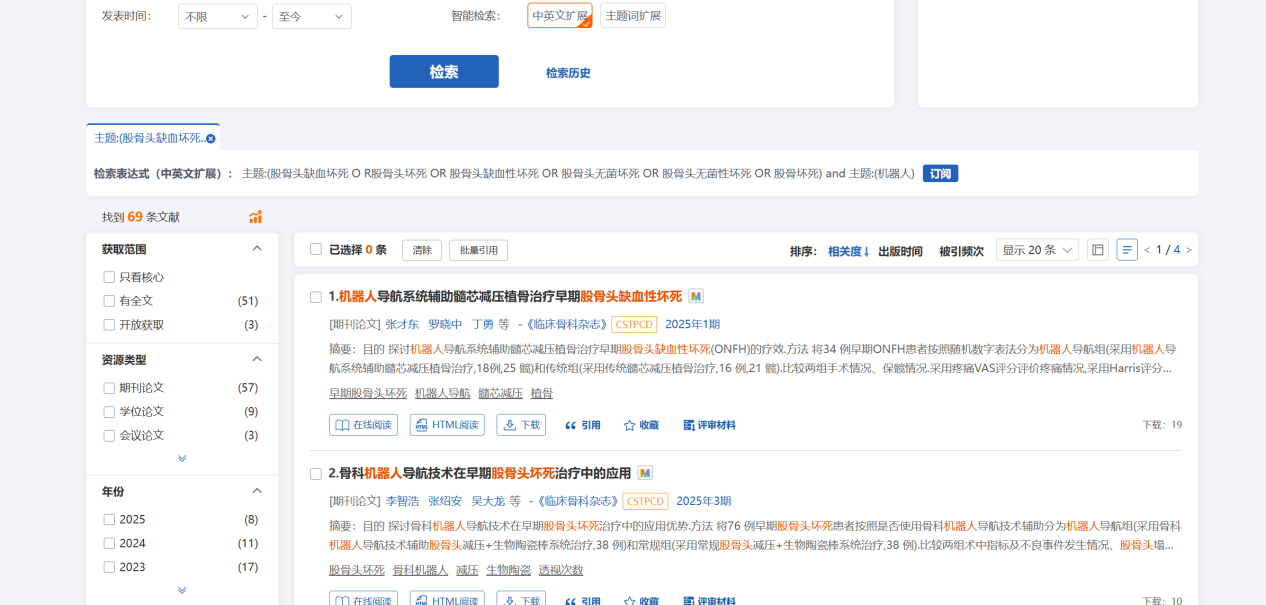


7.VIP data：22 [((((((题名或关键词=股骨头坏死 OR 题名或关键词=股骨头缺血坏死) OR 题名或关键词=股骨头缺血性坏死) OR 题名或关键词=股骨头无菌坏死) OR 题名或关键词=股骨头无菌性坏死) OR 题名或关键词=股骨坏死) AND 题名或关键词=机器人)](https://qikan.cqvip.com/Qikan/search/index?LngMySearHistoryIdGuid=ba53a451-9e73-464c-92ac-3bea8820101d&from=Qikan_Article_History)


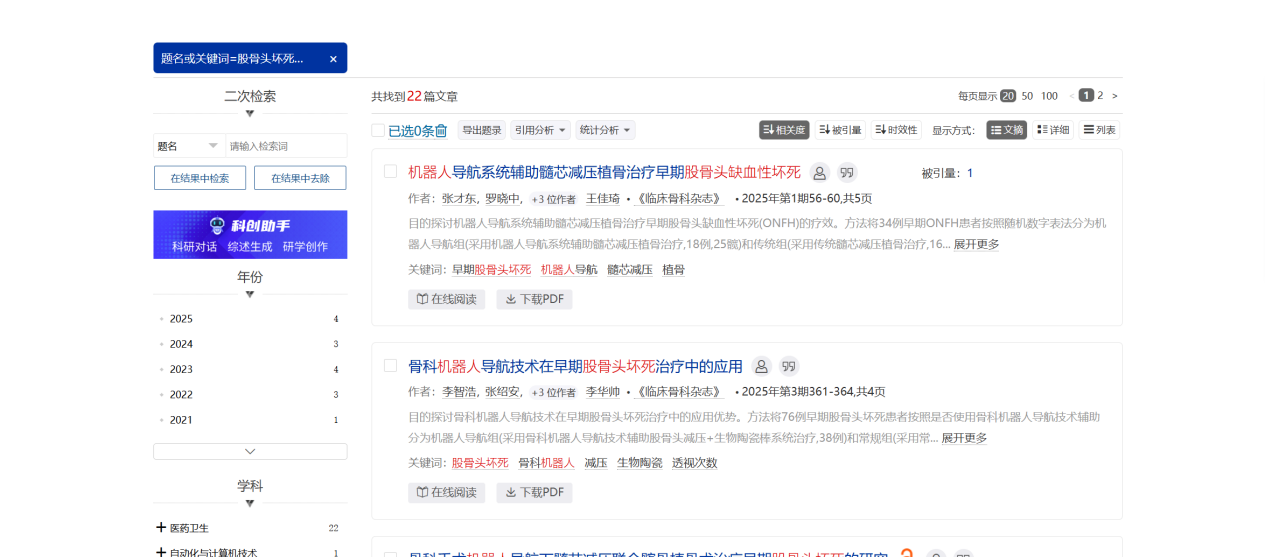


8.CBM: 58 "股骨头缺血坏死OR股骨头坏死OR股骨头缺血性坏死OR股骨头无菌坏死OR股骨头无菌性坏死OR股骨坏死"[常用字段:智能] AND "机器人"[常用字段:智能]
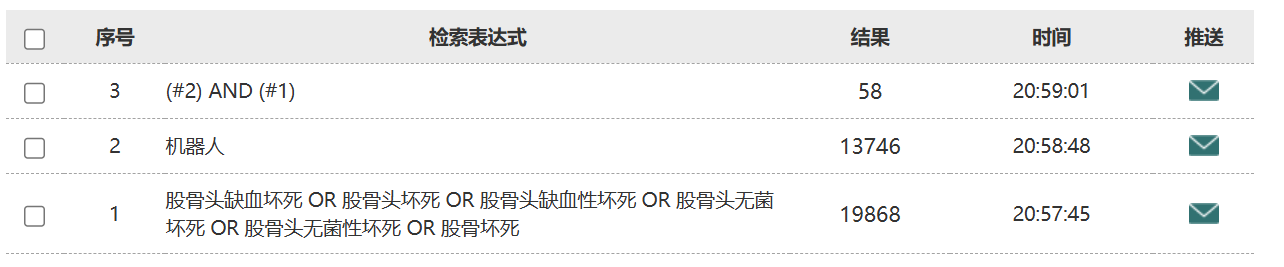


**Supplementary Material 2: Details of duplicate study identification**

| Duplicate studies | the corresponding included studies | Grounds for duplication |
| --- | --- | --- |
| Zhang2021 | Zhai2022 | Consistency in research topic  Consistency in research institution  Consistency in research team |
| Luo2020 | Luo2020 | Consistency in research topic  Consistency in research institution  Consistency in research team |

For duplicate studies, we included the larger sample size and more recently published study in the meta-analysis.

**Supplementary Material 3: Quality Assessment of Included Studies**

Quality Assessment of Cohort Studies

| **Study** | **Selection** | **Comparability** | **Outcome/Exposure** | **Total score** |
| --- | --- | --- | --- | --- |
| Bi2019 | 4 | 1 | 2 | 7 |
| Luo2020 | 4 | 1 | 2 | 7 |
| Zhang2022 | 4 | 1 | 3 | 8 |
| Zhai2022 | 4 | 2 | 2 | 8 |
| Li2023 | 4 | 2 | 3 | 9 |
| Tian2023 | 4 | 1 | 1 | 6 |
| Ma2023 | 4 | 1 | 3 | 8 |
| Cao2024 | 4 | 1 | 3 | 8 |
| Zhang2025 | 4 | 1 | 2 | 7 |
| Kappa value | 1 | 0.526 | 0.842 |  |

Quality Assessment of Case Series

| Study | (1) | (2) | (3) | (4) | (5) | (6) | (7) | (8) | (9) | (10) |
| --- | --- | --- | --- | --- | --- | --- | --- | --- | --- | --- |
| Zhao2018 | Y | Y | Y | U | U | Y | Y | Y | U | Y |
| Yang2022 | Y | Y | Y | U | U | Y | Y | Y | U | Y |
| Kappa value | 1 | 1 | 1 | 1 | 1 | 1 | 1 | 1 | 1 | 1 |

Note: (1) Were there clear criteria for inclusion in the case series?: (2) Was the condition measured in a standard, reliable way for all participants included in the case series?; (3) Were valid methods used for identification of the condition for all participants included in the case series?: (4) Did the case series have consecutive inclusion of participants?: (5) Did the case series have complete inclusion of participants?: (6) Was there clear reporting of the demographics of the participants in the study?: (7) Was there clear reporting of clinical information of the participants?: (8) Were the outcomes or follow up results of cases clearly reported?: (9) Was there clear reporting of the presenting site(s)/clinic(s) demographic information?; (10) Was statistical analysis appropriate? Y: Yes; N: No;U:Unknown

Quality Assessment of Randomized Controlled Trials

| Study | Randomization Process | Deviations from Intended Interventions | Missing Outcome Data | Measurement of the Outcome | Selection of the Reported Result | Overall Risk of Bias |
| --- | --- | --- | --- | --- | --- | --- |
| Zhang2025 | Some concerns | Some concerns | Low risk of bias | Some concerns | Some concerns | Some concerns |
| Kappa value | 1 | 1 | 1 | 1 | 1 |  |

****Supplementary Material 4：Analysis results of different correlation coefficients for HSS and VAS (0.25 and 0.75)****

**Forest plot of the meta-analysis for HSS at a correlation coefficient of 0.25**

****
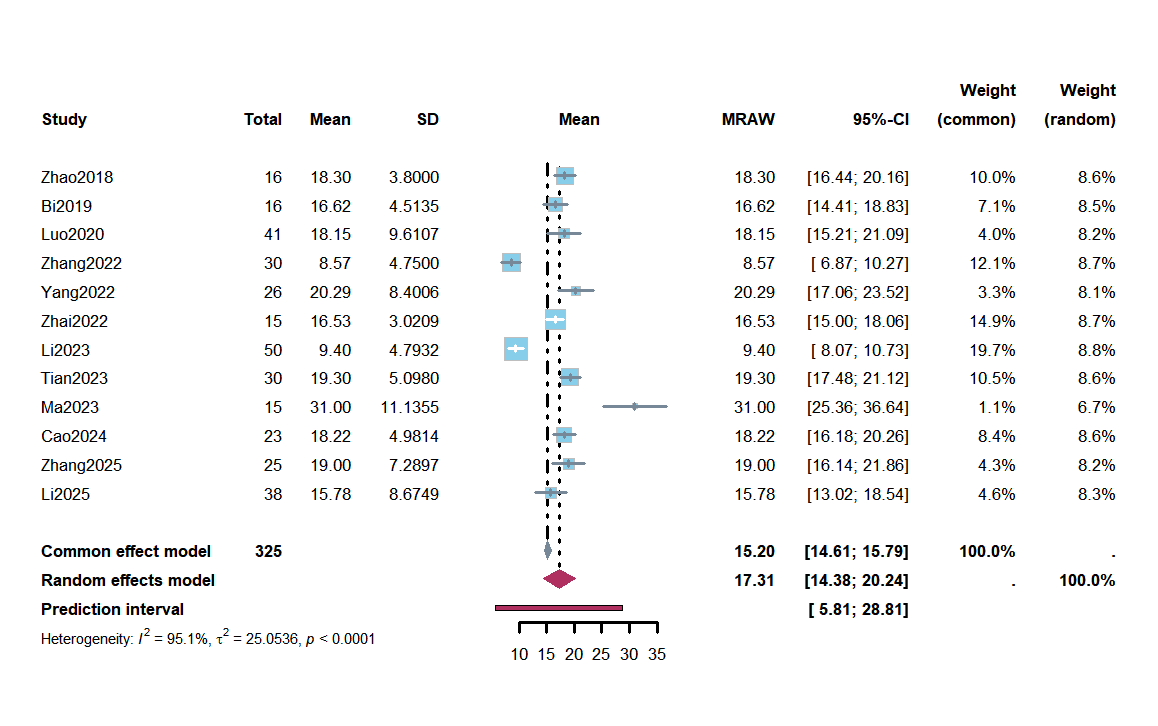
****

**Forest plot of the meta-analysis for HSS at a correlation coefficient of 0.75**

****
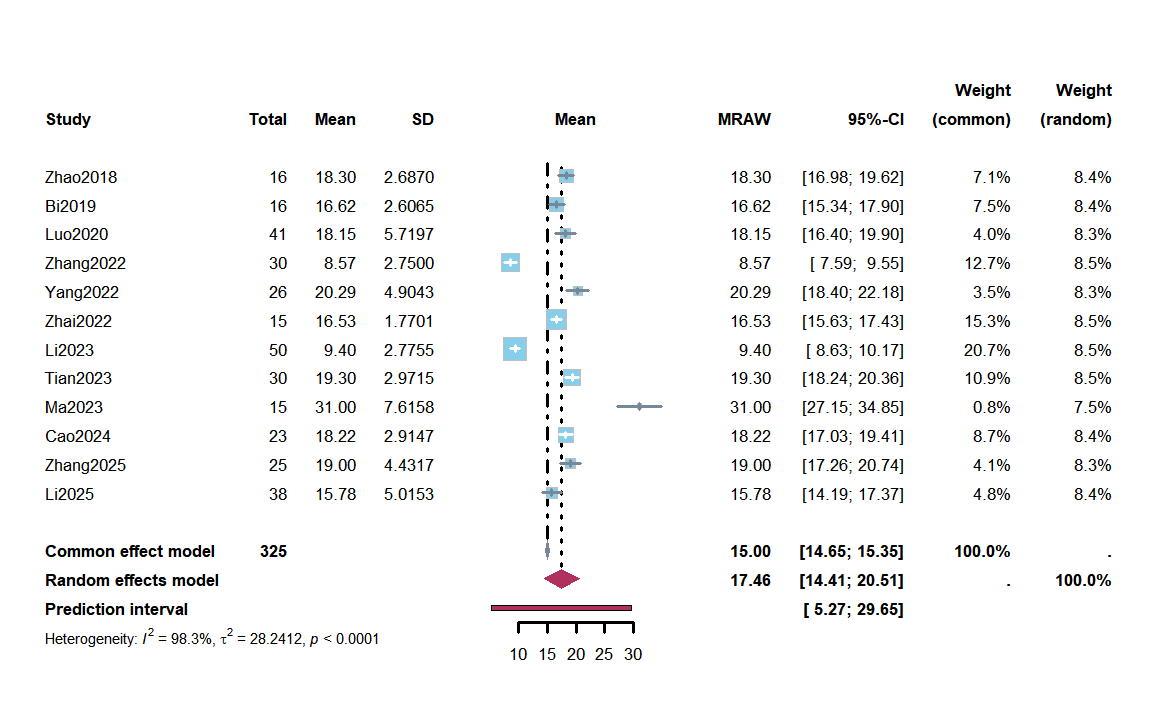
****

**Forest plot of the meta-analysis for VAS at a correlation coefficient of 0.25**

****
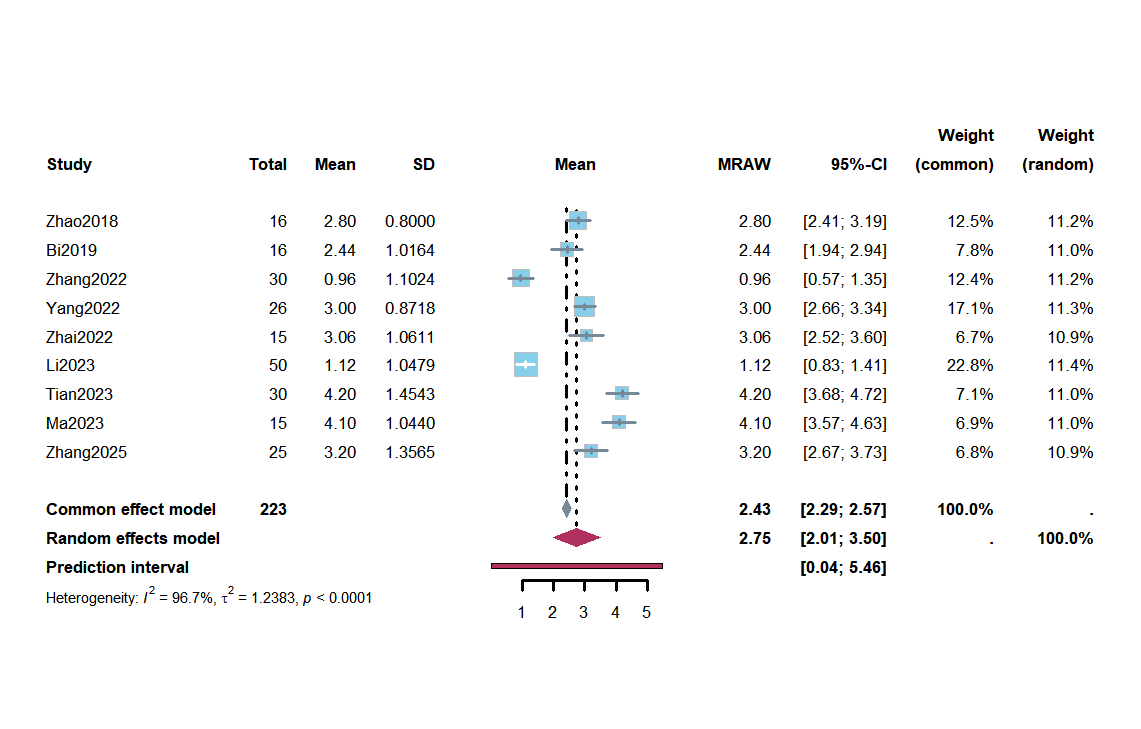
****

**Forest plot of the meta-analysis for VAS at a correlation coefficient of 0.75**

****
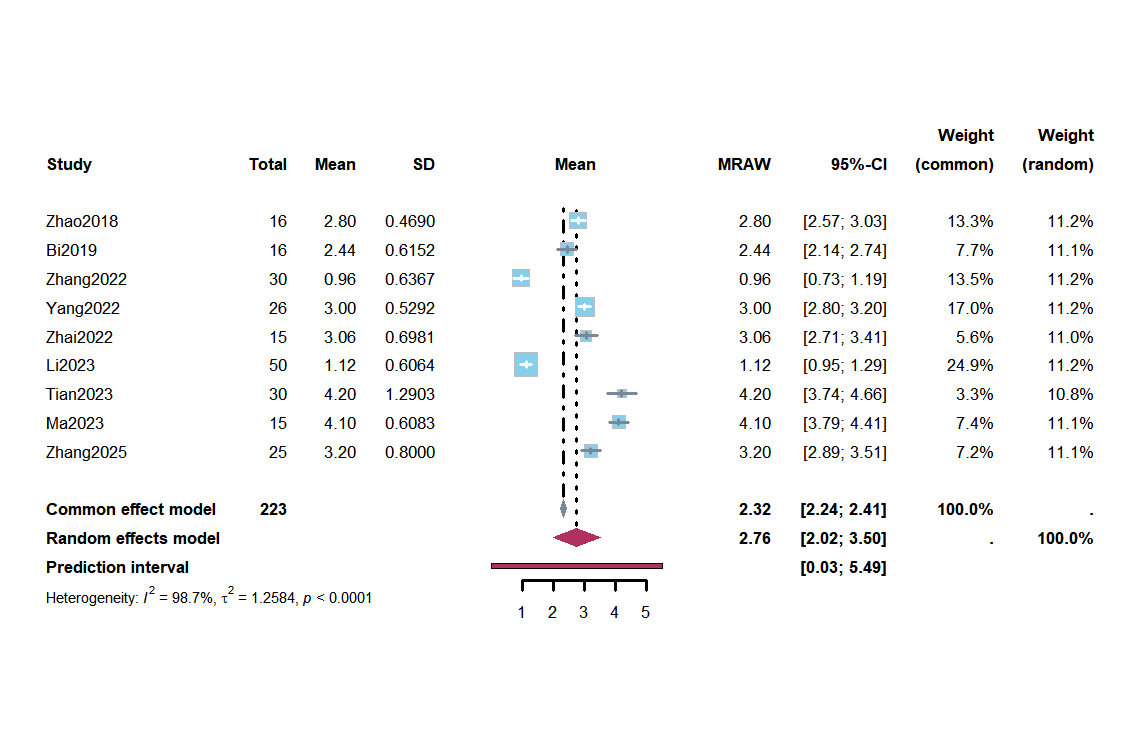
****

****Supplementary Material 5：**Supplementary Analysis on Manual Core Decompression for Femoral Head Collapse Rate Forest Plot**


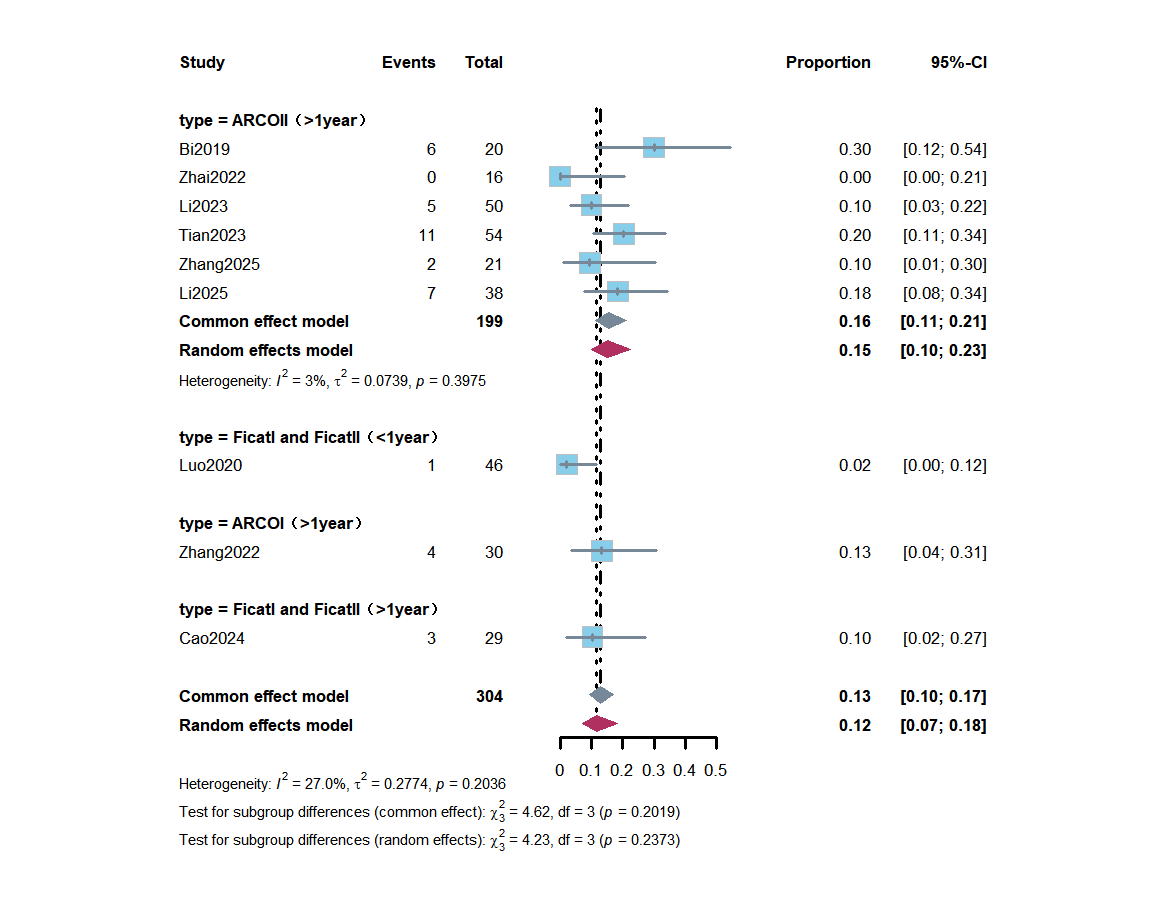


****Forest plot after trim-and-fill adjustment for the femoral head collapse rate in manual core decompression****


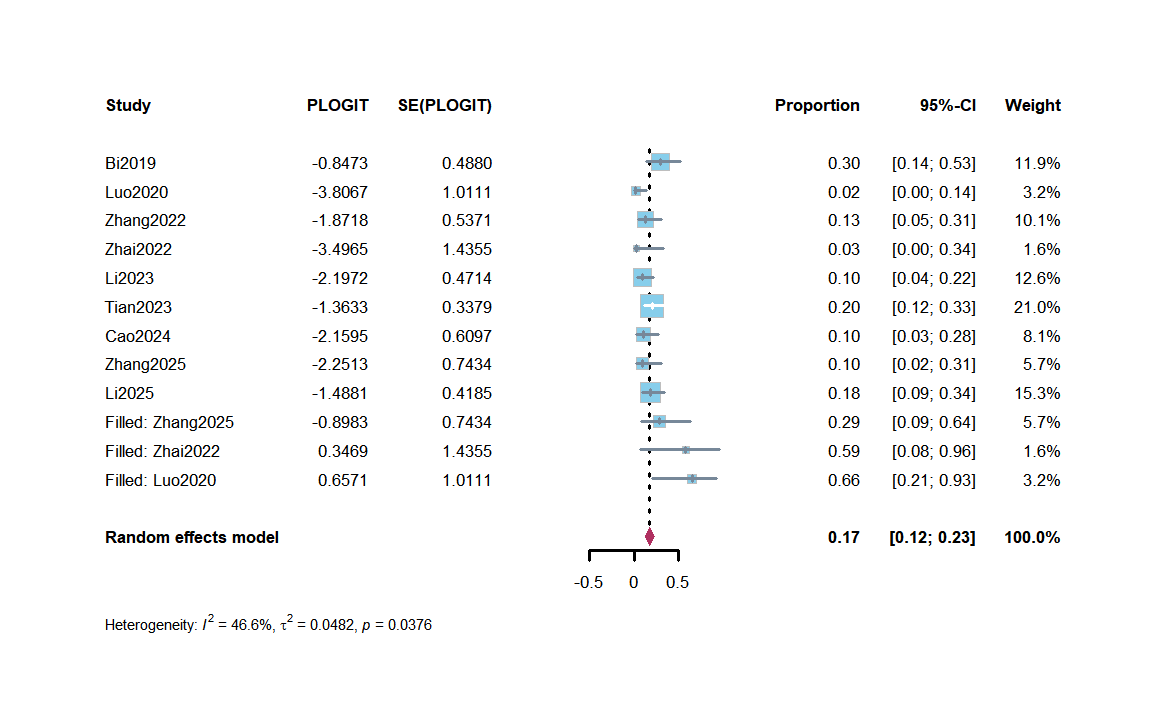


**Head-to-Head Comparison of Subsequent Femoral Head Collapse Risk Between Robot-Assisted and Manual Core Decompression**


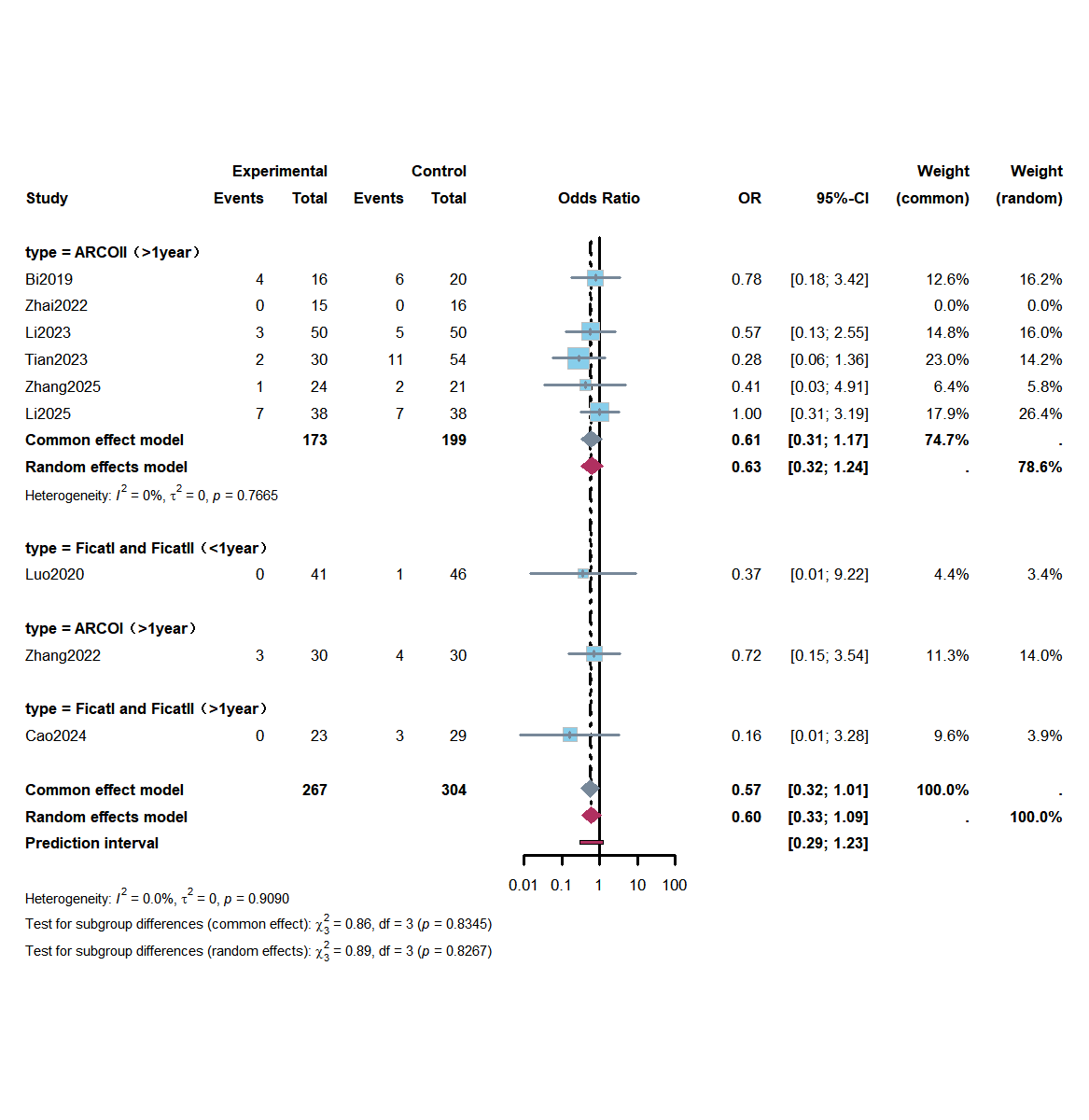


**Supplementary Material 6:Sensitivity Analysis Results**

Sensitivity Analysis of Femoral Head Collapse Rate


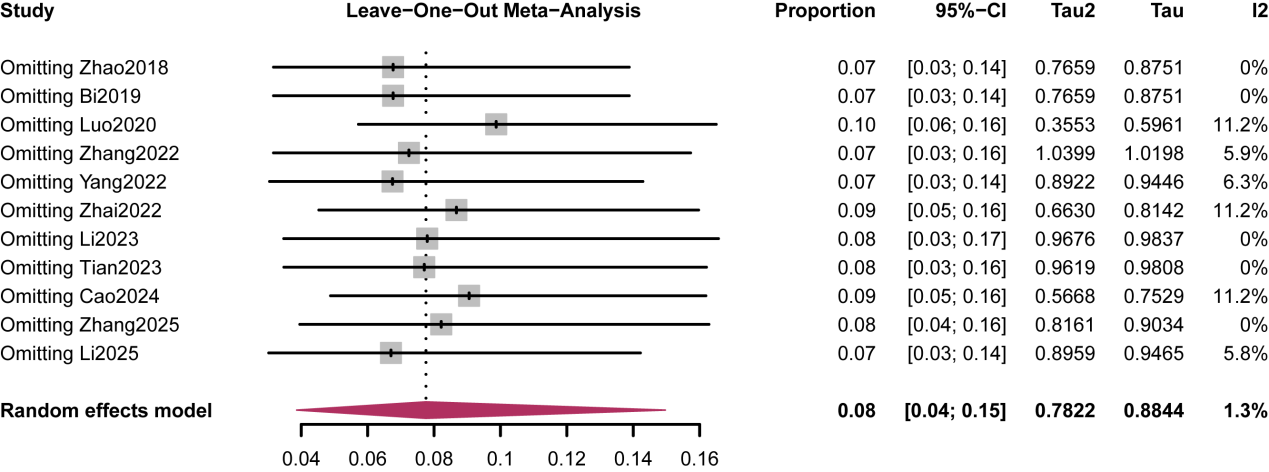


Sensitivity Analysis of HHS


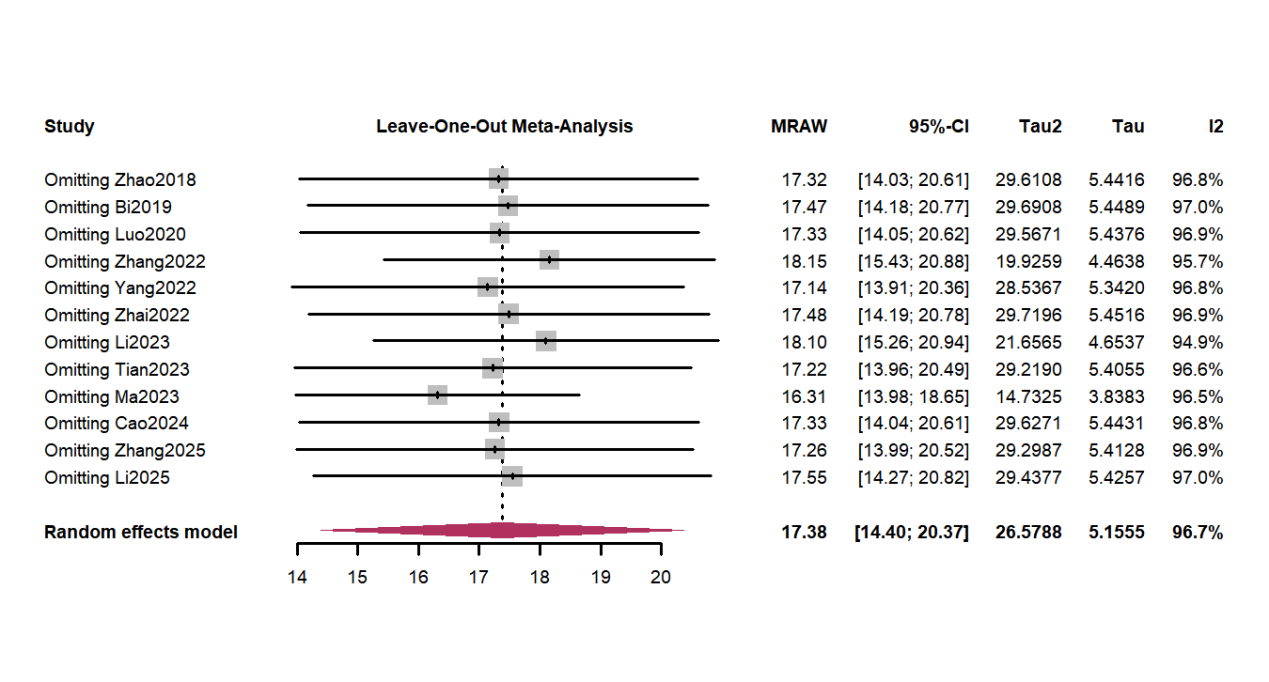


Sensitivity Analysis of VAS


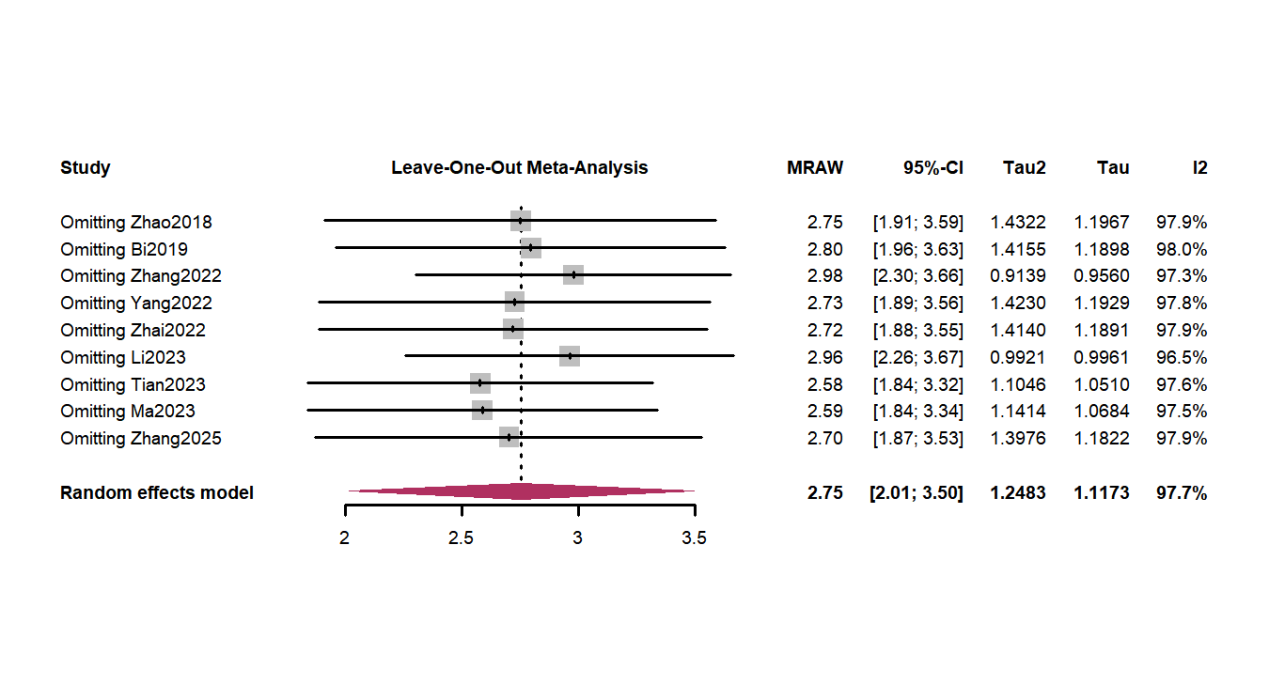


**Supplementary Material 7: The results of the trim-and-fill method for the femoral head collapse rate, HHS and VAS**


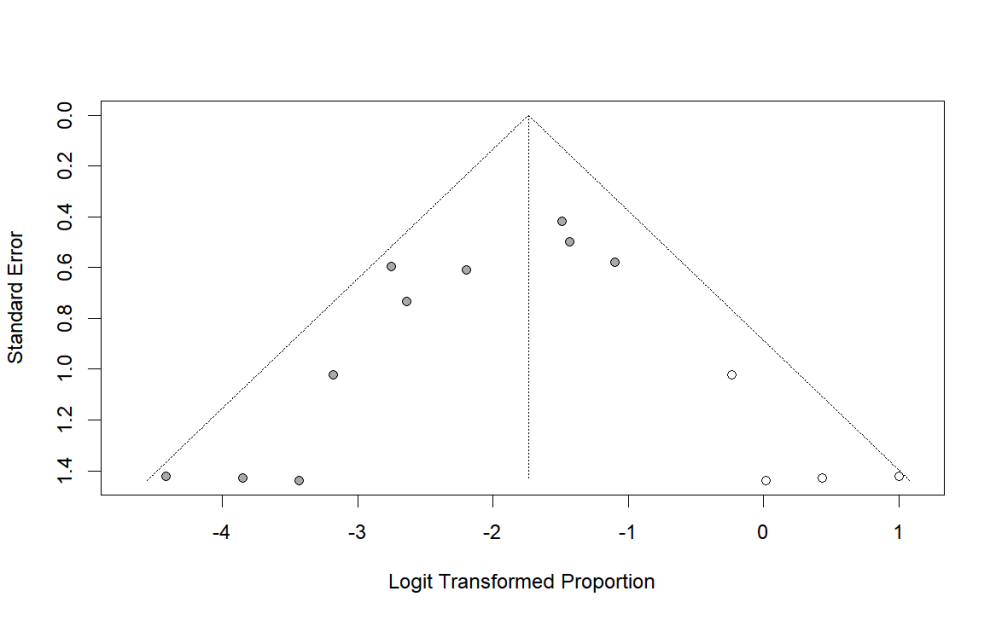
The trim-and-fill funnel plot for the femoral head collapse rate

**The forest plot after trim-and-fill adjustment for the femoral head collapse rate**


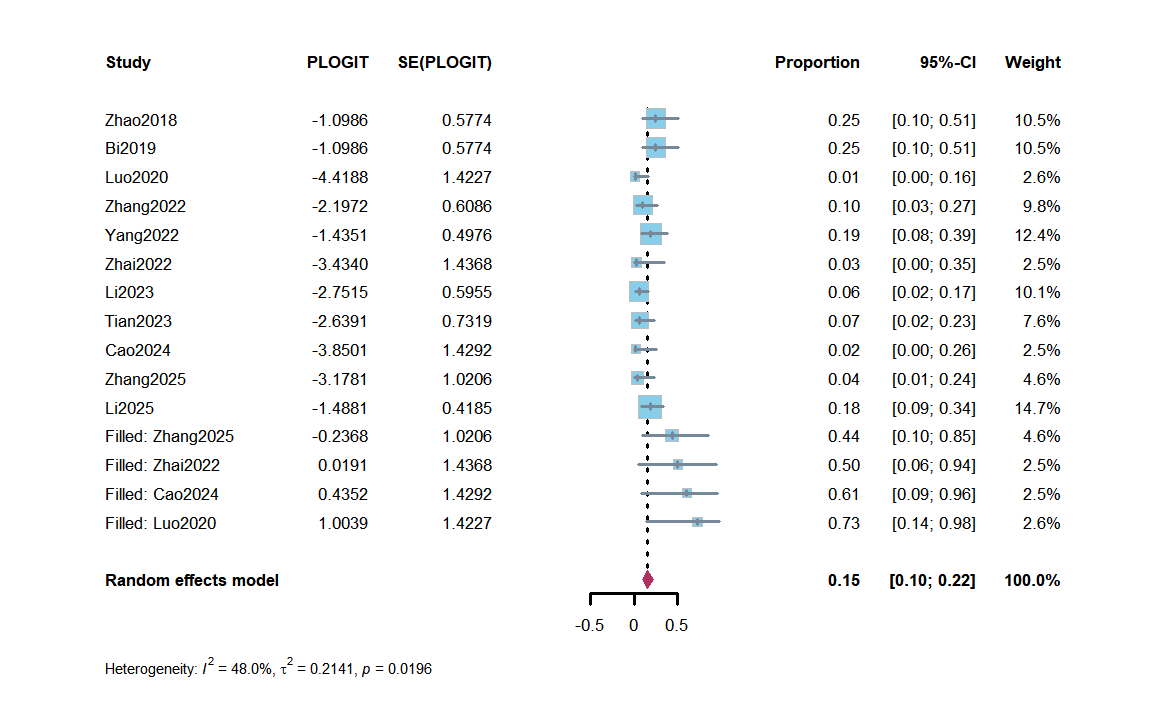


**The trim-and-fill funnel plot for the Harris Hip Score (HHS)**


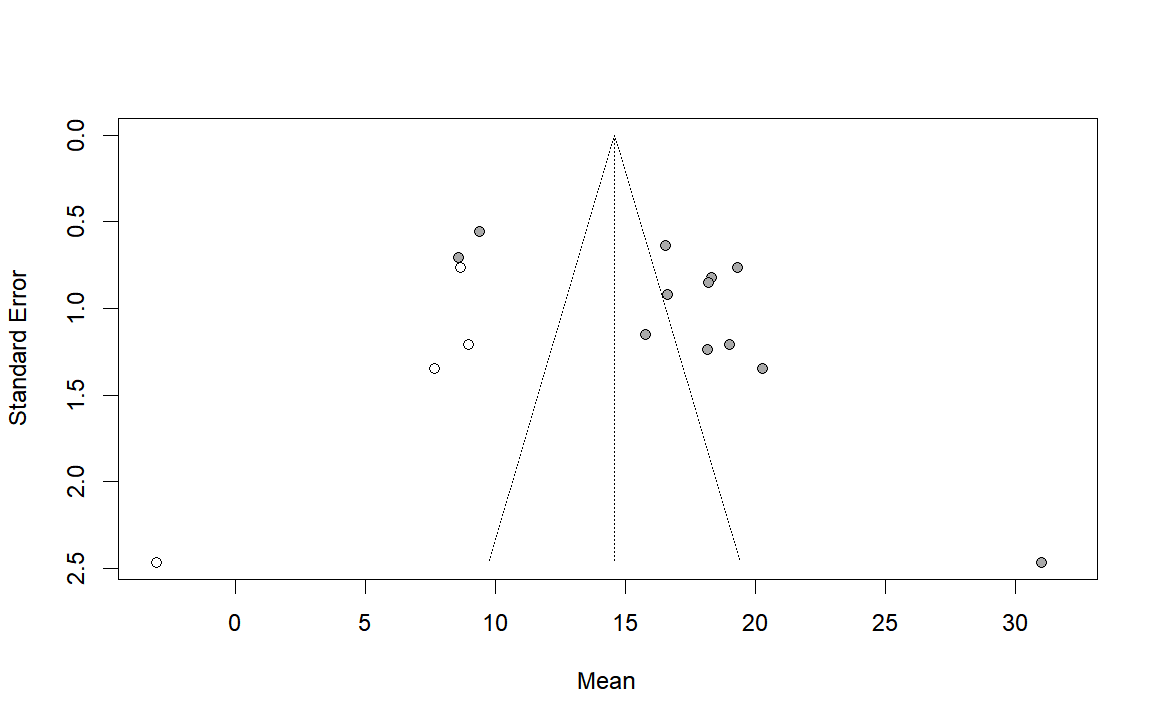


**The forest plot after trim-and-fill adjustment for the Harris Hip Score (HHS)**


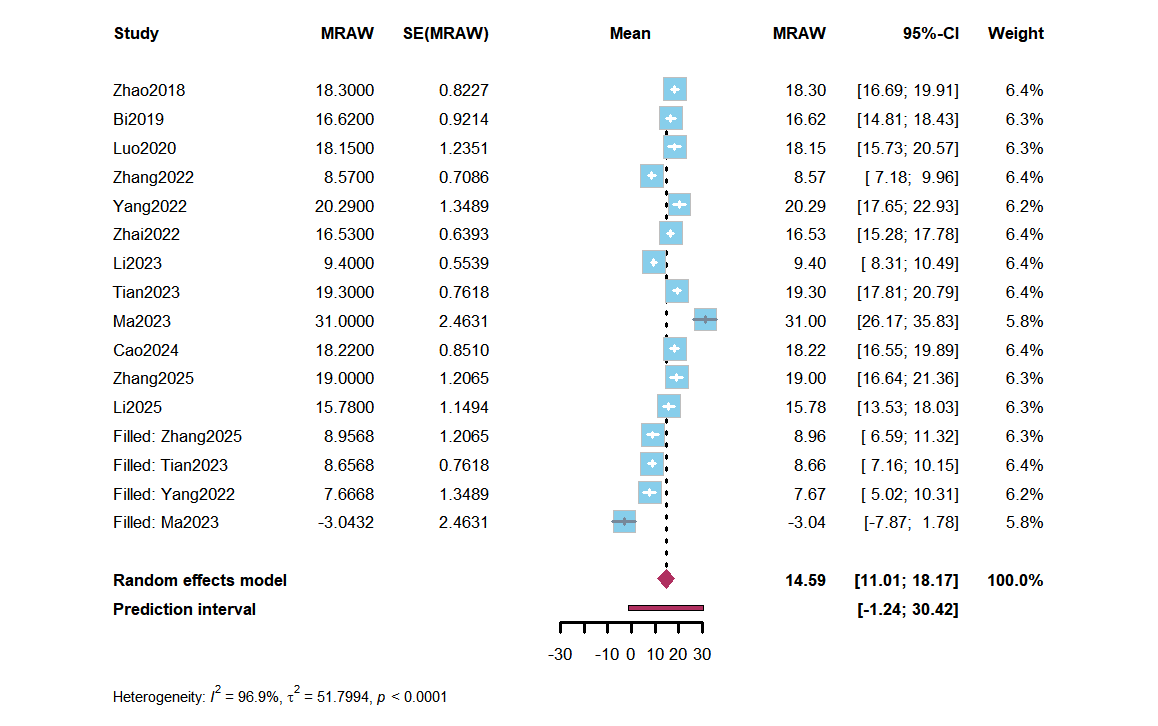


**The trim-and-fill funnel plot for the VAS**

**
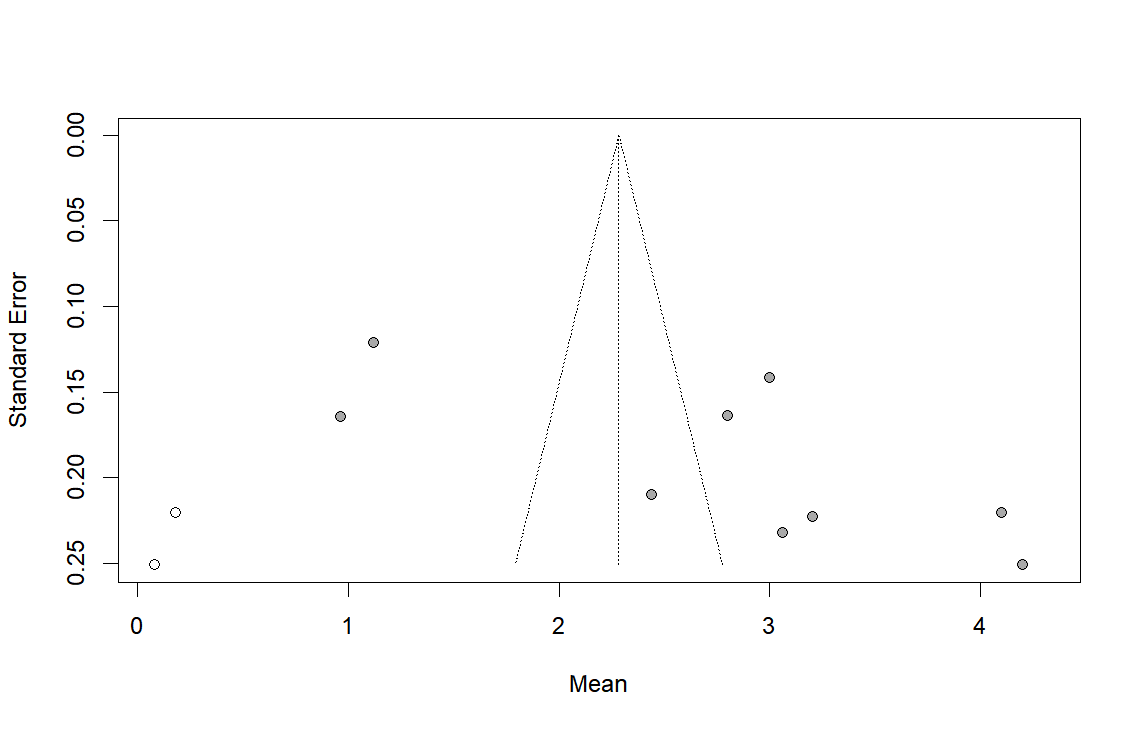
**

**The forest plot after trim-and-fill adjustment for the VAS**

**
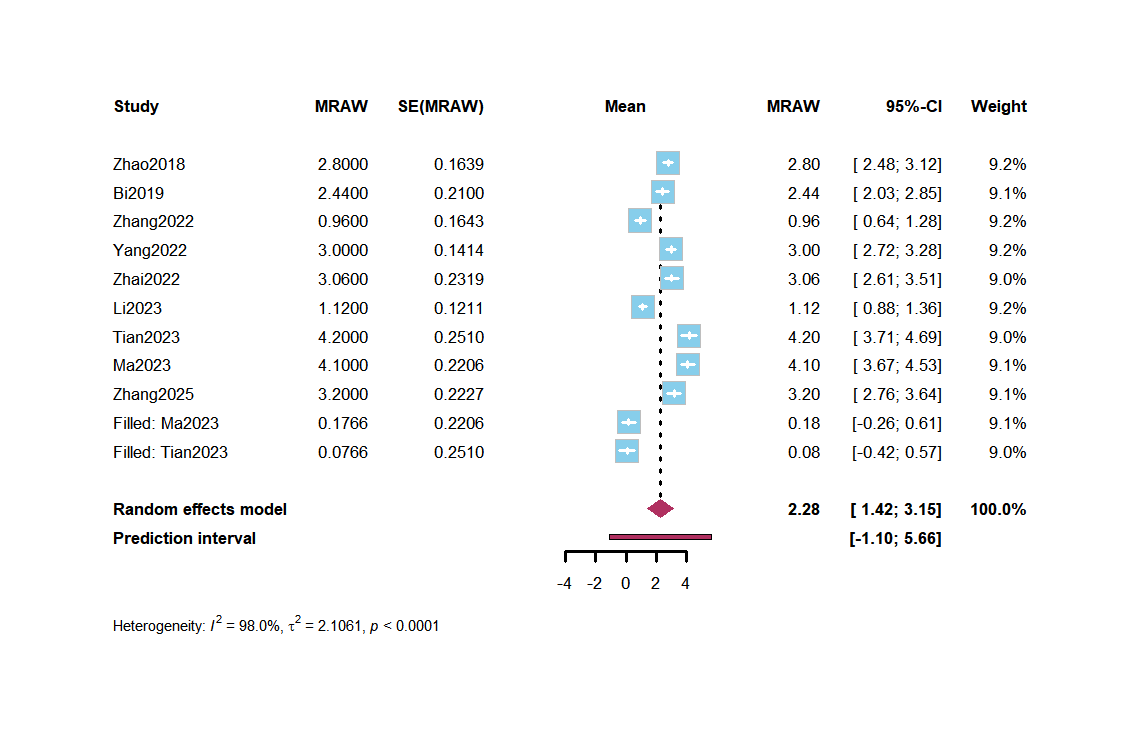
**
